# Coordinated transient interaction of ZO‐1 and afadin is required for pedestal maturation induced by EspF from enteropathogenic *Escherichia coli*

**DOI:** 10.1002/mbo3.931

**Published:** 2019-09-30

**Authors:** Paul Ugalde‐Silva, Fernando Navarro‐Garcia

**Affiliations:** ^1^ Department of Cell Biology Centro de Investigación y de Estudios Avanzados del IPN (CINVESTAV‐IPN) México City Mexico

**Keywords:** actin polymerization, actin rearrangement, afadin, calcium switch, effector protein, Enteropathogenic *E. coli*, EspF, tight junction, TJ disassembly, ZO‐1

## Abstract

Enteropathogenic *Escherichia coli* (EPEC) infection causes a histopathological lesion including recruitment of F‐actin beneath the attached bacteria and formation of actin‐rich pedestal‐like structures. Another important target of EPEC is the tight junction (TJ), and EspF induces displacement of TJ proteins and increased intestinal permeability. Previously, we determined that an EPEC strain lacking EspF did not cause TJ disruption; meanwhile, pedestals were located on the TJ and smaller than those induced by the wild‐type strain. Therefore, EspF could be playing an important role in both phenotypes. Here, using different cell models, we found that EspF was essential for pedestal maturation through ZO‐1 disassembly from TJ, leading to (a) ZO‐1 recruitment to the pedestal structure; no other main TJ proteins were required. Recruited ZO‐1 allowed the afadin recruitment. (b) Afadin recruitment caused an afadin–ZO‐1 transient interaction, like during TJ formation. (c) Afadin and ZO‐1 were segregated to the tip and the stem of pedestal, respectively, causing pedestal maturation. Initiation of these three discrete phases for pedestal maturation functionally and physically required EspF expression. Pedestal maturation process could help coordinate the epithelial actomyosin function by maintaining the actin‐rich column composing the pedestal structure and could be important in the dynamics of the pedestal movement on epithelial cells.

## Graphical abstract

Enteropathogenic Escherichia coli infection causes a histopathological lesion including formation of actin‐rich pedestal‐like structures. We found that EspF is essential for pedestal maturation through ZO‐1 disassembly from the tight junction (TJ): (i) ZO‐1 is recruited to the pedestal structure. Recruited ZO‐1 allows the afadin recruitment. (ii) Afadin recruitment causes an afadin–ZO‐1 transient interaction. (iii) Afadin and ZO‐1 are segregated to the tip and the stem of pedestal, respectively, causing pedestal maturation. Initiation of these three discrete phases for pedestal maturation functionally requires EspF expression.

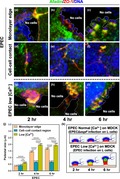

## 1 INTRODUCTION

Enteropathogenic *Escherichia coli* (EPEC) causes a histopathological lesion, attaching and effacing (A/E). This A/E lesion is also caused by other bacterial pathogens, and they are collectively called A/E pathogens, which comprise EPEC, enterohemorrhagic *E. coli* (EHEC), *Citrobacter rodentium*, and *Escherichia albertii*, as well as animal EPEC strains such as rabbit EPEC (REPEC) (Kaper, Nataro, & Mobley, 2004). The features of A/E lesion are localized loss of microvilli and intimate adherence of bacteria to the mammalian cell plasma membrane, followed by recruitment of F‐actin to sites of bacterial attachment. The actin rearrangement ultimately results in the formation of actin‐rich structures called pedestals (Moon, Whipp, Argenzio, Levine, & Giannella, 1983). A/E lesion formation by EPEC requires genes encoded in a 35‐kb chromosomal pathogenicity island designated as the locus of enterocyte effacement (LEE) (McDaniel, Jarvis, Donnenberg, & Kaper, 1995). The LEE encodes components of T3SS, transcriptional regulators, chaperones, and T3SS translocator and effector proteins. Among these effectors, which are translocated directly into host cells, Tir plays an essential role for actin assembly by A/E pathogens (Kenny et al., 1997). After intimin–Tir interaction, the intracellular C‐terminal domain of EPEC Tir is phosphorylated on tyrosine‐474 by mammalian kinases (Bommarius et al., 2007; Phillips, Hayward, & Koronakis, 2004; Swimm et al., 2004). Phosphorylated Tir directly recruits the mammalian adaptor proteins Nck1 and Nck2 (Campellone, Giese, Tipper, & Leong, 2002; Gruenheid et al., 2001; Rohatgi, Nollau, Ho, Kirschner, & Mayer, 2001), which are known activators of the neural Wiskott–Aldrich syndrome protein (N‐WASP)–Arp2/3 pathway for actin assembly in host cells (Kalman et al., 1999; Rohatgi et al., 2001).

Besides Tir, other translocated effectors common to EPEC and related A/E pathogens are also encoded within LEE and injected into the host cell, including Map, EspH, EspF, EspG, and EspZ (Kanack, Crawford, Tatsuno, Karmali, & Kaper, 2005; Matsuzawa, Kuwae, & Abe, 2005; McNamara et al., 2001; Tu, Nisan, Yona, Hanski, & Rosenshine, 2003), as well as less conserved non‐LEE effectors (Dahan et al., 2005; Li et al., 2006; Mundy et al., 2004; Tobe et al., 2006). These LEE or non‐LEE effectors interfere with diverse cell functions. It is worthy to mention that EPEC 2343/69 does not harbor the *E. coli* secreted protein F in prophage U (EspFU) also termed TccP. EspFU is encoded in the O157 island, in contrast to LEE‐encoded EspF (Campellone, Robbins, & Leong, 2004). Moreover, EspFU from canonical EHEC strains is 25% identical to EspF. EspFU displays a unique function because deletion of *espFU* impairs EHEC pedestal formation, whereas deletion of *espF* does not (Campellone et al., 2004; Garmendia et al., 2004), thus implying that these proteins have evolved for distinct cellular functions. Thus, unlike EspF, EspFU is recruited to the pedestal and is associated indirectly with Tir, since Tir from canonical EHEC strains (O157:H7) does not have the residue Y474 (Campellone et al., 2004). On the other hand, EspF is clearly involved with another important target of EPEC, the tight junction (TJ) complex, which leads to the displacement of several TJ proteins and increased permeability through the intestinal epithelium (Dean & Kenny, 2009). Besides the disruption of the epithelial barrier, EspF has been localized in multiple cellular compartments (including cytoplasm, mitochondria, nucleolus, and apical and lateral membranes) and interacts with at least 12 reported host proteins. Once delivered, EspF is associated with mitochondrial dysfunction, destruction of the nucleolus, microvilli effacement, tight junction disruption, apoptosis, epithelial transporter inhibition, antiphagocytosis, vesicular trafficking manipulation, membrane remodeling, and actin‐pedestal maturation (Alto et al., 2007; Dean & Kenny, 2004; Guttman et al., 2006; Hodges, Alto, Ramaswamy, Dudeja, & Hecht, 2008; Nagai, Abe, & Sasakawa, 2005; Nougayrede & Donnenberg, 2004; Peralta‐Ramirez et al., 2008; Shaw, Cleary, Murphy, Frankel, & Knutton, 2005). It is believed that its multifunctional behavior relies on the presence of specific motifs since EspF contains an N‐terminal mitochondrial targeting signal (amino acids 1–24), a nucleolus targeting signal (amino acids 21–74), and three proline‐rich repeats (PRR) at the C‐terminus (Holmes, Muhlen, Roe, & Dean, 2010).

We have shown that EspF from EPEC E2348/69 has three almost identical proline‐rich sequences, which can be recognized by class I SH3 domains, and three class III PDZ domain binding motifs (Peralta‐Ramirez et al., 2008). In eukaryotic cells, these motifs are relevant for protein–protein interaction, that is, actin regulator proteins containing SH3 domains, and motifs interacting with PDZ domains present in scaffolding factors that recruit signaling molecules to cell junctions, including the zonula occludens‐1 (ZO‐1), ZO‐2, and ZO‐3 junctional proteins (Peralta‐Ramirez et al., 2008). Thus, these EspF proline‐rich motifs and PDZ domain binding motifs might be related to actin rearrangement and TJ disruption. In agreement with these in silico predictions, we also showed that after 2 hr of infection, EspF bound to the N‐WASP and Arp2/3, as well as ZO‐1 and ZO‐2 proteins (Peralta‐Ramirez et al., 2008). In fact, it has been shown that N‐WASP regulates the apical junction complex homeostasis and that EspF exploits both N‐WASP and SNX9 to disrupt intestinal barrier integrity during infection (Garber et al. 2017).

The actin cytoskeleton and the scaffold proteins are key for tight junctions’ integrity. TJs are mainly composed of transmembrane proteins such as occludin, claudins, JAMs, and tricellulin, which are associated with the cytoplasmic plaque formed by ZO‐1/2/3, connecting tight junction to the actin cytoskeleton, and cingulin and paracingulin connecting TJ to the microtubule network (Ugalde‐Silva, Gonzalez‐Lugo, & Navarro‐Garcia, 2016). ZO‐1 regulates the permeability through the modulation of the actin cytoskeleton (Van Itallie, Fanning, Bridges, & Anderson, 2009; Zihni, Mills, Matter, & Balda, 2016). F‐actin is required for formation and maintenance of TJs and adherens junctions (AJs), and afadin, an F‐actin binding protein localized at the AJs, regulates the formation of AJs and TJs. During the formation of AJs, afadin–nectin first recruits JAMs and then occludin and claudin through the interaction of afadin with ZO‐1 for the formation of TJs (Sakakibara, Maruo, Miyata, Mizutani, & Takai, 2018). In this context, we have found two interesting phenomena: An EPEC strain lacking EspF did not cause TJ disruption and recruitment of TJ proteins into the pedestal, and pedestals were smaller than those induced by the wild‐type strain; the latter were located mainly on the TJ (Peralta‐Ramirez et al., 2008). Thus, EspF interaction with host proteins induces the recruitment of junctional proteins into the pedestals, leading to the maturation of actin pedestals and paracellular permeability disruption. We speculated that the pedestal maturation could be important not only for the initial colonization but also for bacterial spreading by influencing the dynamics of the pedestal movement along and between epithelial cells. In order to understand how EspF could be influencing the pedestal maturation and its relationship with TJ proteins, we infected different cell lines as well as known polarized cells under the calcium switch assay and performed confocal microscopy, co‐immunoprecipitation, and knockdown assays for understanding the role of the tight junction proteins and to decipher the mechanism involved in pedestal maturation induced by EspF‐producing EPEC during epithelial cell infection.

## 2 RESULTS

### 2.1 Disassembly of cell junction proteins is related to pedestal growth induced by EPEC

**Figure A1:**
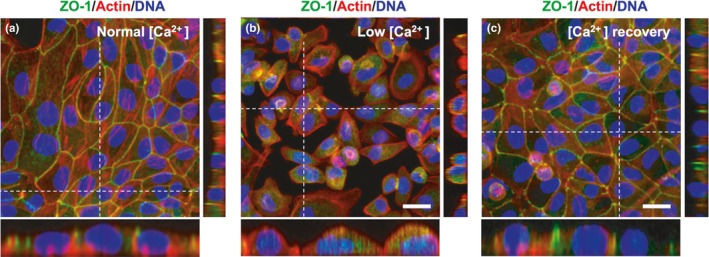
A calcium switch model in MDCK cells to analyze assembly–disassembly–assembly of intercellular junctions. MDCK cells were seeded at 95% of confluency and then subjected to calcium switch assay: (a) MDCK cells were kept in DMEM containing normal concentration of calcium, without antibiotics and bovine fetal serum (BFS) for 6 hr. (b) MDCK cells were washed and kept in fresh DMEM containing low concentration of calcium, without antibiotics and bovine fetal serum (BFS) for 6 hr. (c) MDCK cells were treated as in panel (b) and then the medium with low concentration of calcium was removed and it was replaced by DMEM containing normal concentration of calcium and incubated for another 2 hr. Cells under the different conditions were fixed, permeabilized, and stained with DAPI to detect nuclear DNA and FAS (rhodamine–phalloidin) for F‐actin fibers. The stained cells were immunostained with a rabbit anti‐ZO‐1 polyclonal antibody followed by a secondary antibody, FITC‐goat anti‐rabbit. Slides were analyzed and recorded by confocal microscopy (63× zoom 1). Each panel shows maximum projection and two *z*‐sections (*zx* and *zy*). Bar: 20 µm

**Figure A2:**
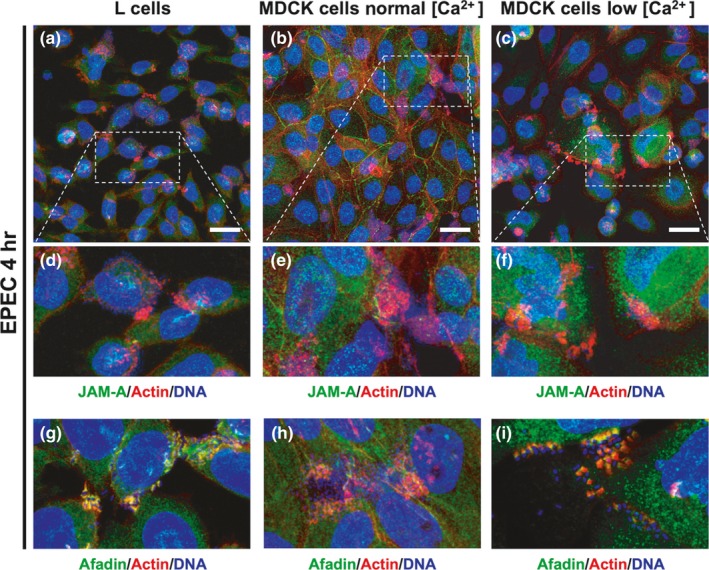
JAM is not recruited in the pedestal structure at 4 hr of infection when afadin and ZO‐1 are dissociated into this structure. L or MDCK cells were infected with EPEC at a MOI of 0.5 for 4 hr. Cells were fixed, permeabilized, and stained with DAPI to detect bacterial and nuclear DNA (blue) and FAS (rhodamine–phalloidin) for F‐actin (red). The stained cells were immunostained with a rabbit anti‐JAM‐A (a‐f) or anti‐afadin (g–i) polyclonal antibody followed by a biotin‐SP‐conjugated AffiniPure goat anti‐rabbit IgG and then fluorescein‐conjugated streptavidin (green). (a, d, and g) EPEC‐infected L cells. (b, e, and h) EPEC‐infected MDCK at normal calcium concentration and (c, f, and i) EPEC‐infected MDCK at low calcium concentration. Unlike afadin (arrows), JAM‐A is not recruited to the pedestal structures. Bar: 20 µm

We previously found that EspF is involved in intercellular junction disassembly and ZO‐1 recruitment to pedestals formed by REPEC E22 (Peralta‐Ramirez et al., 2008). Thereby, we decided to investigate in detail the role of TJ proteins in the increase of the pedestal size. We used the model of calcium switch using MDCK cells. In this model, at normal calcium concentration MDCK cells formed mature monolayers with assembled TJs, which were clearly detected using anti‐ZO‐1 antibodies. These antibodies decorated a continuous pattern of ZO‐1 distribution along the cell periphery marking the cell–cell junction with the classical chicken wire staining (Figure A1a). But when calcium was removed from the cell medium, the TJs were disassembled and the junctional proteins were dispersed in the cytoplasm as detected by ZO‐1 immunolabeling (Figure A2b). Once calcium was added back to the medium, the cells initiated the formation of TJs (Figure A1c) once again, as before calcium removal (see Figure A1a). Monolayers under these three modalities were also observed in *z*‐slices, where clearly ZO‐1 was detected at the TJ position in normal‐calcium (Figure A1a) and calcium‐recovery (Figure A1c) cells, but not in low‐calcium cells, where ZO‐1 was found dispersed in the cytoplasm (Figure A1b).

**Figure 1:**
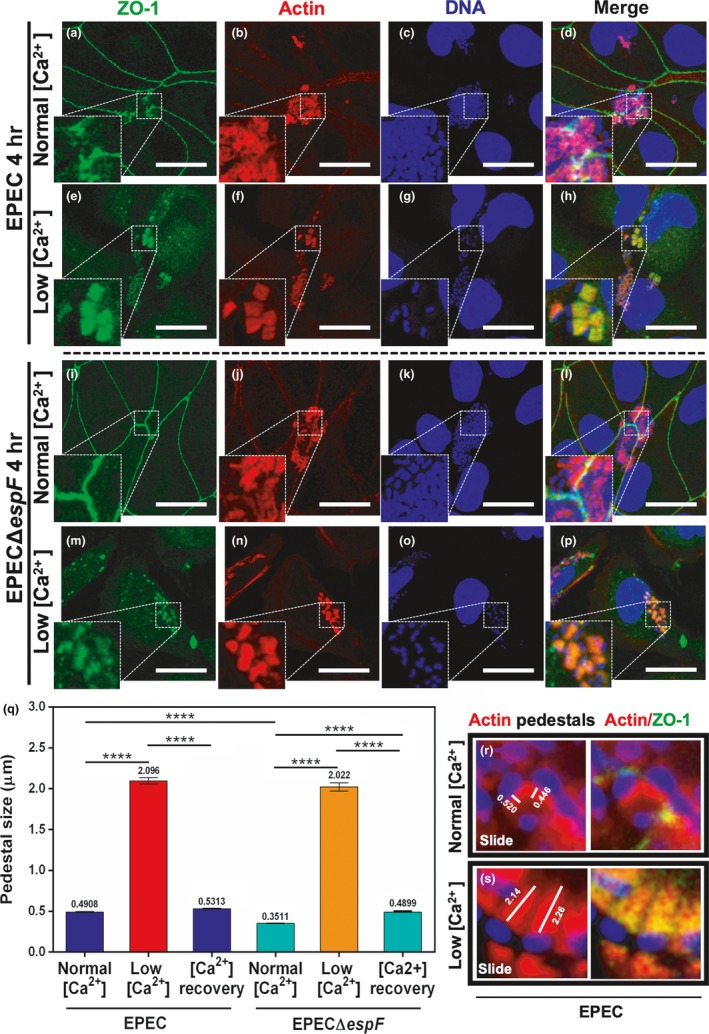
Disassembly of cell–cell junctions by using the calcium switch model or by EspF promotes the growth of pedestals. MDCK cells were infected with EPEC wild type (a–d) or EPECΔ*espF* (i–l) at a MOI of 0.5 for 4 hr (normal calcium concentration). Two other groups of cells were infected: in cells kept in DMEM containing low concentration of calcium (TJ disassembly condition) for 2 hr, those with EPEC (e–h) or EPECΔ*espF* (m–p), and in cells returned to normal concentration of calcium for 2 hr after incubation at low calcium concentration (calcium recovery) (see panel q). Cells were fixed, permeabilized, and stained with DAPI (for bacterial and nuclear DNA) and FAS (rhodamine–phalloidin for F‐actin). The stained cells were immunostained with a rabbit anti‐ZO‐1 polyclonal antibody followed by a secondary antibody, FITC‐goat anti‐rabbit IgG. Slides were analyzed and recorded by confocal microscopy (63X zoom 3). Bar: 20 µm. From each panel, sections of 0.5 μm were used to measure individual pedestals, 150 from three independent experiments (i.e., R and S are sections from panels d and h). (q) Pedestals were measured (µm) using the Leica Lite software, and data were plotted and analyzed using GraphPad Prism 6.0. All data from the different strains were compared with EPECΔ*espF* using a one‐way ANOVA test, *n* = 3 independent experiments. **p < .005, ****p < .0001

Thus, by using the calcium switch model, we compared the size of the actin pedestals induced by EPEC between cells forming cell junctions (normal calcium) or cells with disassembled junctional proteins (low calcium) infected with either EPEC E2348/69 or EPECΔ*espF* for 4 hr. At normal calcium concentration, most of the actin pedestals formed by either EPEC or EPECΔ*espF* were detected mainly on the intercellular junctions (Figure 1a–d and i–l). Interestingly, in the case of cells infected by EPEC, ZO‐1 signal was discontinued and undetected right where the bacteria were forming the pedestal structure, whereas the same signal appeared continuous along intercellular junction in sites devoid of bacteria (Figure 1a–d). In contrast, cells infected by EPECΔ*espF* retained a continuous ZO‐1 distribution pattern, which was not interrupted by the presence of the actin pedestals adhered on the intercellular junctions, even though the intercellular junctions appeared to be under stress (Figure 1i–l). These data indicate and support previous data showing that EspF promotes the disassembly of ZO‐1 from the TJs (see zoom in Figure 1a vs. i). Additionally, MDCK cells infected with any of the two strains under normal calcium concentration exhibited pedestals with slight recruited ZO‐1 labeling, in contrast to epithelial cells of low transepithelial electrical resistance (TER) or without TER, such as Caco/B7 or HeLa cells (Hanajima‐Ozawa et al., 2007). Interestingly, at normal calcium concentration, the pedestals formed by EPEC were significantly bigger (0.49 µm) than those formed by EPECΔ*espF* (0.35 µm) (Figure 1q). On the other hand, at low calcium concentration, the intercellular junctions of MDCK cells were disassembled and ZO‐1 labeling was mainly detected in the cytoplasm. Infection with wild‐type EPEC (Figure 1e–h) or EPECΔ*espF* (Figure 1m–p) under low calcium concentration induced bigger pedestals than those in cells grown in medium with a normal calcium concentration, and the ZO‐1 signal was also detected inside the pedestals (Figure 1h, p). However, while labeling for both ZO‐1 and F‐actin coincided inside the pedestals of cells infected by wild‐type EPEC (Figure 1h), this pattern occurred in less extension in cells infected by EPECΔ*espF* (Figure 1p) and the pedestals were formed mainly on the cell periphery (Figure 1n vs. f). Nevertheless, disassembly of junctional proteins due to low calcium concentration caused that pedestals induced by either wild‐type EPEC or EPECΔ*espF* were of the same size, around 4 times higher (EPEC 2.09 µm and EPECΔ*espF* 2.02 µm) than those formed in normal‐calcium conditions (Figure 1q). Measurement of pedestals in Figure 1q was facilitated by using optical sections and confocal software; pedestals were easily detected as those shown in Figure 1r and Figure 1s. ZO‐1 labeling in MDCK cells with restored normal calcium concentration was detected again in the continuous pattern along the restored intercellular junctions, being interrupted only where the bacteria were forming the pedestals (data not shown). Interestingly, pedestals formed by any of the strains in the condition of calcium recovery were reduced in size (0.5313 µm for EPEC and 0.4899 µm for EPECΔ*espF*), and they were not statistically different (Figure 1q). Taken together, these data indicate that EspF presence during the infection induces ZO‐1 disassembly from TJs. Furthermore, ZO‐1 availability in the cytoplasm due to TJ disassembly (induced by EspF or by calcium switch) is important to increase the pedestal size.

### 2.2 ZO‐1 is necessary and enough to increase the pedestal size

**Figure 2:**
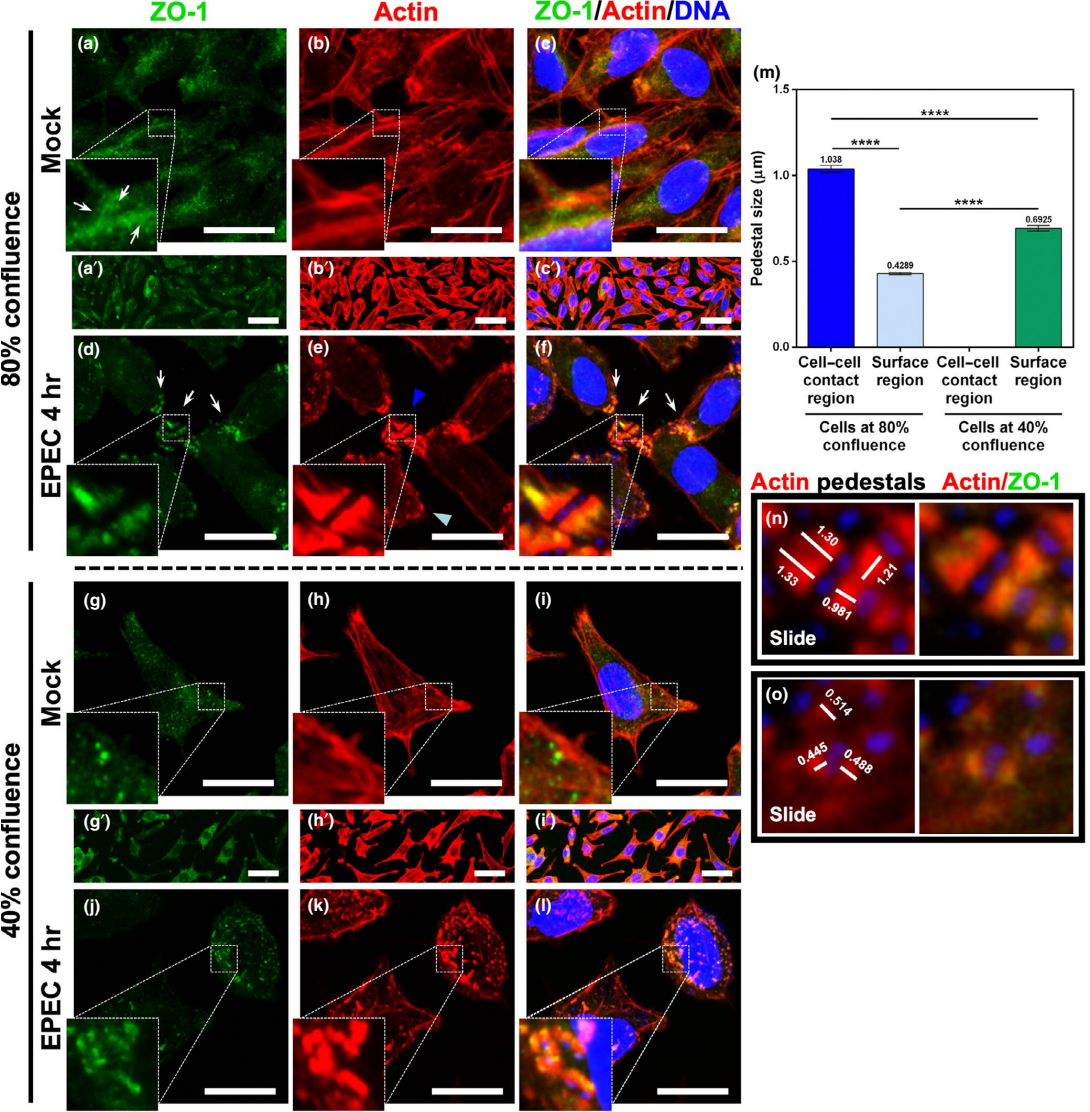
ZO‐1 is necessary and sufficient to cause an increase of pedestal size by EspF‐expressing EPEC, without other main junctional proteins. L cells were grown at 80% (a–f) and 40% (g–l) of confluence. L cells without infection were used as mock cells at 80% (a–c, and at low magnification, a′–c′) and 40% (g–i, and at low magnification, g′–i′) of confluence. Cells at 80% (d–f) and 40% (j–l) of confluence were infected with EPEC wild type at a MOI of 0.5 for 4 hr. Cells were fixed, permeabilized, and stained with DAPI (blue) and FAS (red) as mentioned in Figure 1. The stained cells were immunostained with a rabbit anti‐ZO‐1 polyclonal antibody followed by a secondary antibody, FITC‐goat anti‐rabbit IgG. Slides were analyzed and recorded by confocal microscopy (63X zoom 3). Bar: 20 µm. Arrows point out ZO‐1 puncta and arrowheads the size of pedestals in cell–cell interaction (blue) and cell surface region (cyan). From each panel, sections of 0.5 μm were used to measure individual pedestals, 150 from three independent experiments (i.e., [n] and [o] are sections from panels [f] and [l]). (m) Pedestals at the different confluences, as well as those on the cell–cell contacts or on the cell surface regions, were measured (µm) using the Leica Lite software, and data were plotted and analyzed using GraphPad Prism 6.0. Data were compared using a one‐way ANOVA test, *n* = 3 independent experiments. ****p < .0001

To understand the role of the different TJ proteins, such as claudin, occludin, or tricellulin, we used the fibroblast line, L cells, which do not form intercellular junctions. L cells do not contain any of the intercellular junction proteins mentioned above but ZO‐1 and can be used to transfect them for expressing other junctional proteins. First, we determined ZO‐1 distribution in noninfected semiconfluent L cells (80%). At this confluency (Figure 2a′–c′), ZO‐1 was found mainly in the cytoplasm, but interestingly, accumulation of immunolabeled ZO‐1 puncta was detected in some regions of cell–cell contacts (Figure 2a–c). Then, we infected L cells at this confluency with EPEC. Intriguingly, EPEC was able to form pedestals to which ZO‐1 was recruited, despite the lack of expression of important TJ proteins in these cells, such as claudin and occludin. Interestingly, two different types of pedestal populations were observed: a minority of small pedestals formed at the cell surface and a majority of large pedestals. The latter were formed in the regions of cell–cell contacts, where a higher number of ZO‐1 puncta accumulation was present (Figure 2d–f).

Since the cell–cell contact regions, enriched in ZO‐1, promoted the pedestal growth, we decided to grow L cells at a confluency that would avoid the formation of cell–cell contacts. L cells were grown at 40% confluency (Figure 2g′–i′), and ZO‐1 was immunodetected in EPEC‐infected cells. At this confluency (subconfluent L cells), cells did not form contacts and ZO‐1 puncta were not detected. In contrast, ZO‐1 labeling revealed an exclusively disperse pattern in the cytoplasm (Figure 2g–i). After the infection with EPEC, the pedestals were smaller than those formed at cell–cell contacts of semiconfluent cells despite the recruitment of ZO‐1 to actin pedestals. It is noteworthy that infected subconfluent cells presented a dispersed pattern of pedestals on their cell surface (Figure 2j–l).

To evaluate the effect of cell–cell contact formation on the genesis of pedestals, we measured the pedestal size of the different populations (Figure 2m) formed at both confluency levels (Figure 2n and O). Pedestals formed at the cell–cell contact regions of infected semiconfluent cells were significantly larger (1.038 µm) than those observed elsewhere on the cell surface (0.429 µm). In contrast, large pedestals were not detected on the cell surface of infected subconfluent cells. Subconfluent cells displayed scattered pedestals that were larger than those of the semiconfluent cells (0.6925 µm); however, these pedestals were smaller than cell–cell contact pedestals observed for semiconfluent cells (Figure 2m). These data indicate that ZO‐1 availability (disassembled from TJs) in the cytoplasm is important for inducing the growth of pedestals. On the other hand, since L cells do not express relevant junctional proteins such as claudin, occludin, ZO‐2, and ZO‐3, our data suggest that no other relevant proteins from the TJs might be required for this process. These data also suggest that other proteins in the cell–cell contact that recruit ZO‐1 to these sites could also be involved in the pedestal growth.

### 2.3 EspF is required for sequential recruiting of ZO‐1 and afadin to pedestals induced by EPEC

**Figure 3:**
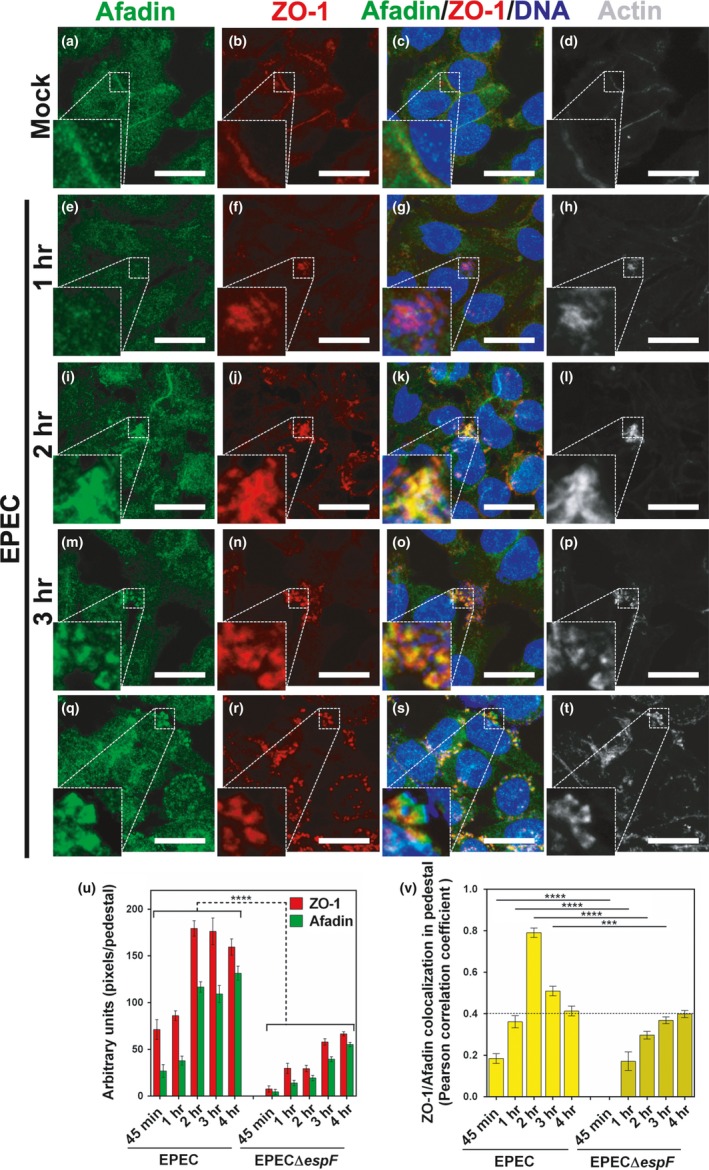
Afadin and ZO‐1 transiently colocalize in the pedestal‐like structure induced by EPEC. L cells were grown at 80% of confluence in mock conditions (a–d) and infected with EPEC at a MOI of 0.5 at different times: 1 (e–h), 2 (i–l), 3 (m–p), or 4 hr (q–t). Cells were fixed, permeabilized, and stained with DAPI (DNA, blue) (c, g, k, o, s) and FAS (F‐actin, pseudocolored gray) (d, h, l, p, t). The stained cells were immunostained with a mouse anti‐ZO‐1 monoclonal antibody followed by a secondary antibody, CY5‐donkey anti‐mouse IgG (pseudocolored red) (b, f, j, n, r), and with a rabbit anti‐afadin polyclonal antibody followed by a biotin‐SP‐conjugated AffiniPure goat anti‐rabbit IgG and then fluorescein‐conjugated streptavidin (green) (a, e, i, m, q). Slides were analyzed and recorded by confocal microscopy (63X zoom 3). Each panel is projecting a zoom for showing actin pedestals beneath adhered bacteria. Bar: 20 µm. (u) Recruitment of ZO‐1 and afadin into the pedestal‐like structure. Red (ZO‐1) or green (afadin) pixels into each pedestal (150 in total) were measured using the Fiji 2.1.0 software (ImageJ) at the different infection times. (v) Colocalization of ZO‐1 and afadin into the pedestal‐like structure. Pearson's correlation coefficient was performed using ImageJ, and data of colocalization by pedestal were plotted to compare both strains. The dotted line indicates the value (0.4) considered as less than moderate values of colocalization according to a Fuzzy Linguistic System. Data were compared using a one‐way ANOVA test, *n* = 3 independent experiments. ***p < .0005; ****p < .0001

Afadin is a regulator of intercellular junction assembly in epithelial cells. Through its interaction with the TJ protein ZO‐1 and adherent junction protein α‐catenin, afadin regulates the assembly of TJs and AJs (Birukova et al., 2012). To determine the role of afadin, as a possible partner protein of ZO‐1 in the pedestal growth, we compared the cell localization of afadin in uninfected L cells and infected with EPEC using an anti‐afadin antibody. In uninfected cells, the detection of afadin was relatively weak in cell–cell contacts (Figure 3a), and the detection of ZO‐1 was similarly weak in the same zones of cell–cell contacts (Figure 3b), but both signals lacked colocalization (Figure 3c). L cells were infected with EPEC following a time course of 45 min, 1, 2, 3, and 4 hr. At 45 min of infection, afadin signal disappeared from the cell–cell contacts and was detected mainly distributed in the cytoplasm, whereas ZO‐1 began to be recruited to the pedestal primordia coinciding with F‐actin signal beneath the EPEC microcolony (data not shown). At 1 hr of infection, afadin was not clearly detected into the actin pedestals (Figure 3e), unlike ZO‐1 that was accumulated in these structures, but no colocalization between afadin and ZO‐1 was detected (Figure 3g). At 2 hr of infection, increasing ZO‐1 signal was detected in these cumuli in the pedestal structures (Figure 3j), and surprisingly, afadin was abundantly detected in the actin pedestals (Figure 3i), strongly colocalizing with ZO‐1 in these structures (Figure 3k), but only in the highest pedestals and not in the smaller one. At 3 hr of infection, the colocalization signal between afadin (Figure 3m) and ZO‐1 (Figure 3n) began to disappear as individual signals started to segregate inside of the largest pedestals (Figure 3o), while in the pedestals of smaller size, the afadin–ZO‐1 colocalization remained constant, or only ZO‐1 was detected (Figure 3p, O, and N). Remarkably, at 4 hr of infection, the signals for afadin and ZO‐1 in the largest pedestals appeared separated while adopting a unique distribution pattern, where afadin labeling was located at the tip of pedestals beneath adhered bacteria whereas ZO‐1 signal was detected in the stem of pedestals. Meanwhile within smaller pedestals, afadin and ZO‐1 signals remained colocalized or alternatively revealed a signal for ZO‐1 only (Figure 3s). Interestingly, using the same system for afadin labeling but using anti‐JAM‐A as a first antibody, we could not detect JAM‐A in the pedestals nor associated with ZO‐1 in these structures at 4 hr of infection of L or MDCK cells (Figure A2), which was different than during the TJ formation, where JAM‐A is associated with ZO‐1 after its dissociation from afadin (Ooshio et al., 2010).

**Figure A3:**
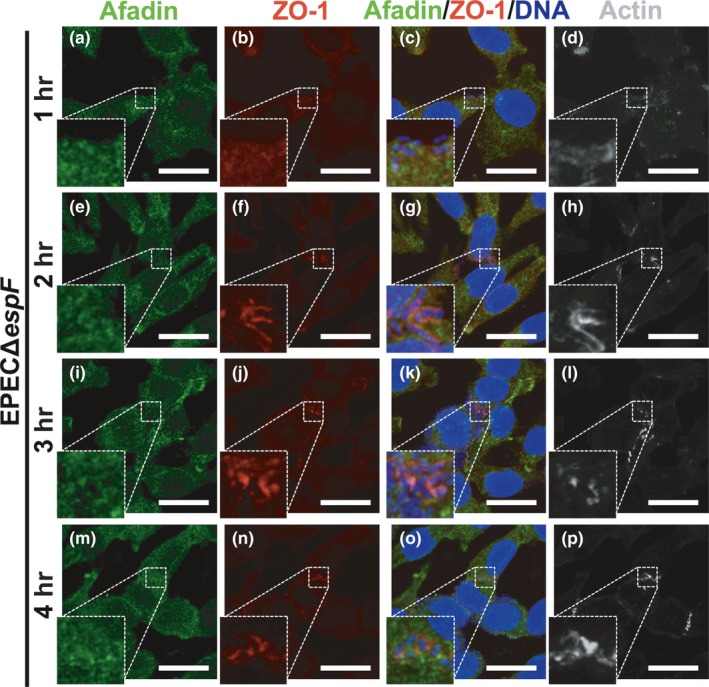
Afadin and ZO‐1 do not colocalize in the pedestal‐like structure induced by EPECΔ*espF*. L cells were grown at 80% of confluence and infected with EPECΔ*espF* at a MOI of 0.5 at different times 1 (a–d), 2 (e–h), 3 (i–l), or 4 hr (m–p). Cells were fixed, permeabilized, and stained with DAPI to detect bacterial and nuclear DNA (blue) (c, g, k, o) and FAS (rhodamine–phalloidin) for F‐actin fibers (pseudocolored gray) (d, h, l, p). The stained cells were immunostained with a mouse anti‐ZO‐1 monoclonal antibody followed by a secondary antibody, CY5‐donkey anti‐mouse IgG (pseudocolored red) (b, f, j, n), and with a rabbit anti‐afadin polyclonal antibody followed by a biotin‐SP‐conjugated AffiniPure goat anti‐rabbit IgG and then fluorescein‐conjugated streptavidin (green) (a, e, i, p). Slides were analyzed and recorded by confocal microscopy (63X zoom 3). Each panel is projecting a zoom for showing actin pedestals beneath adhered bacteria. Bar: 20 µm

To gain insights into the role of EspF in the dynamics of ZO‐1 and afadin recruitment to the pedestal structures, L cells were infected with EPECΔ*espF* for 45 min, 1, 2, 3, or 4 hr. At 45 min of infection, actin‐pedestal primordia were not detected while both ZO‐1 staining and afadin staining were detected in the cytoplasm as dispersed signals, which did not colocalize (data not shown). At 1 hr of infection, actin pedestals remained undetected (Figure A3d), whereas afadin (Figure A3a) and ZO‐1 (Figure A3b) signals retained their distribution in the cytoplasm as dispersed signals that were not colocalized (Figure A3c). At 2 hr of infection, afadin labeling remained scattered in the cytoplasm (Figure A3e), whereas ZO‐1 was detected in the very small actin‐pedestal structures (almost lineal) (Figure A3f, h). These evidences support our previous observations, which revealed that infection with EPEC that lacked *espF* led to the formation of smaller pedestals than those in wild‐type EPEC infections. Notably, at 3 and 4 hr of infection, the afadin signal remained undetectable in the pedestal structures (Figure A3i, m) while the recruited ZO‐1 signal decorated the small actin pedestals (Figure A3j–l and n–p) but with less signal intensity than that observed in wild‐type EPEC‐induced pedestals (Figure A3j, n). Interestingly, there was a complete absence of colocalization between afadin and ZO‐1 in the pedestals and these were smaller in size at all infection times analyzed with EPECΔ*espF.*

To acquire a better understanding of the kinetics of ZO‐1 and afadin recruitment to the pedestal structures, we quantified the signal intensity of both proteins in 150 pedestals during the infection kinetics with either EPEC or EPECΔ*espF* strains (Figure 3u). The signal intensity of ZO‐1 recruitment to the pedestals significantly increased with the infection time, from the start of the pedestal formation at 45 min and 1 hr (around 70 pixels per pedestal) until 2 hr of infection (around 180 pixels per pedestal), to then remain stable signal at 3 and 4 hr of infection. Interestingly, ZO‐1 recruitment values to the pedestals in EPECΔ*espF*‐infected cells were drastically decreased in comparison with the values by the wild‐type EPEC infection, at every infection time point, from the start of pedestal formation, which was delayed with respect to the wild type (1 and 2 hr, ~30 pixels/pedestal), until 3 and 4 hr of infection (around 60 pixels per pedestal). In the case of afadin, the recruitment to the EPEC‐induced pedestals was very poor at the start of pedestal at 45 min and 1 hr of infection (around 30 pixels per pedestal) (Figure 3u). Afadin recruitment, values became noticeable at 2 hr of infection (around 120 pixels per pedestal) and plateaued at 4 hr of infection. On the other hand, the infection with the mutant EPECΔ*espF* caused a drastic decrease in afadin recruitment at every infection time points, in comparison with EPEC‐infected cells, with very low values of 5 pixels per pedestal at 45 min of infection reaching a maximum of 50 pixels per pedestal at 4 hr of infection, which could be considered background levels of detection (Figure 3u). These data strongly suggest that EPEC can recruit first ZO‐1 to the actin pedestals (about 1 hr) followed by afadin (about 2 hr) and that this sequential recruitment depends on EspF expression.

Based on our result that ZO‐1 and afadin were transitory colocalized into the pedestals that peaked at 2 hr and gradually decreased until 4 hr of the infection by EPEC, we decided to quantify this phenomenon using Pearson's correlation coefficient analysis of cells infected by EPEC or EPECΔ*espF* (Figure 3v). In the case of pedestals formed by EPEC, no colocalization was detected at 45 min or 1 hr of infection; however, ZO‐1 and afadin colocalization suddenly increased at 2 hr of infection. This colocalization decreased at 3 hr and went undetected at 4 hr of infection, coinciding with the separation of ZO‐1 and afadin in the stem and tip pattern of the previously described large pedestals. In contrast, EPECΔ*espF*‐induced pedestals revealed a low colocalization coefficient value between afadin and ZO‐1 throughout the total duration of the infection time course. Taken together, these data strongly suggest that the process of pedestal growth and maturation comprises three phases: first, ZO‐1 recruitment to the pedestal structures (small pedestals); then, afadin recruitment and colocalization with ZO‐1 (medium pedestals); and finally, separation of these two proteins into the pedestal in a stem and tip pattern, respectively (big pedestals).

**Figure 4:**
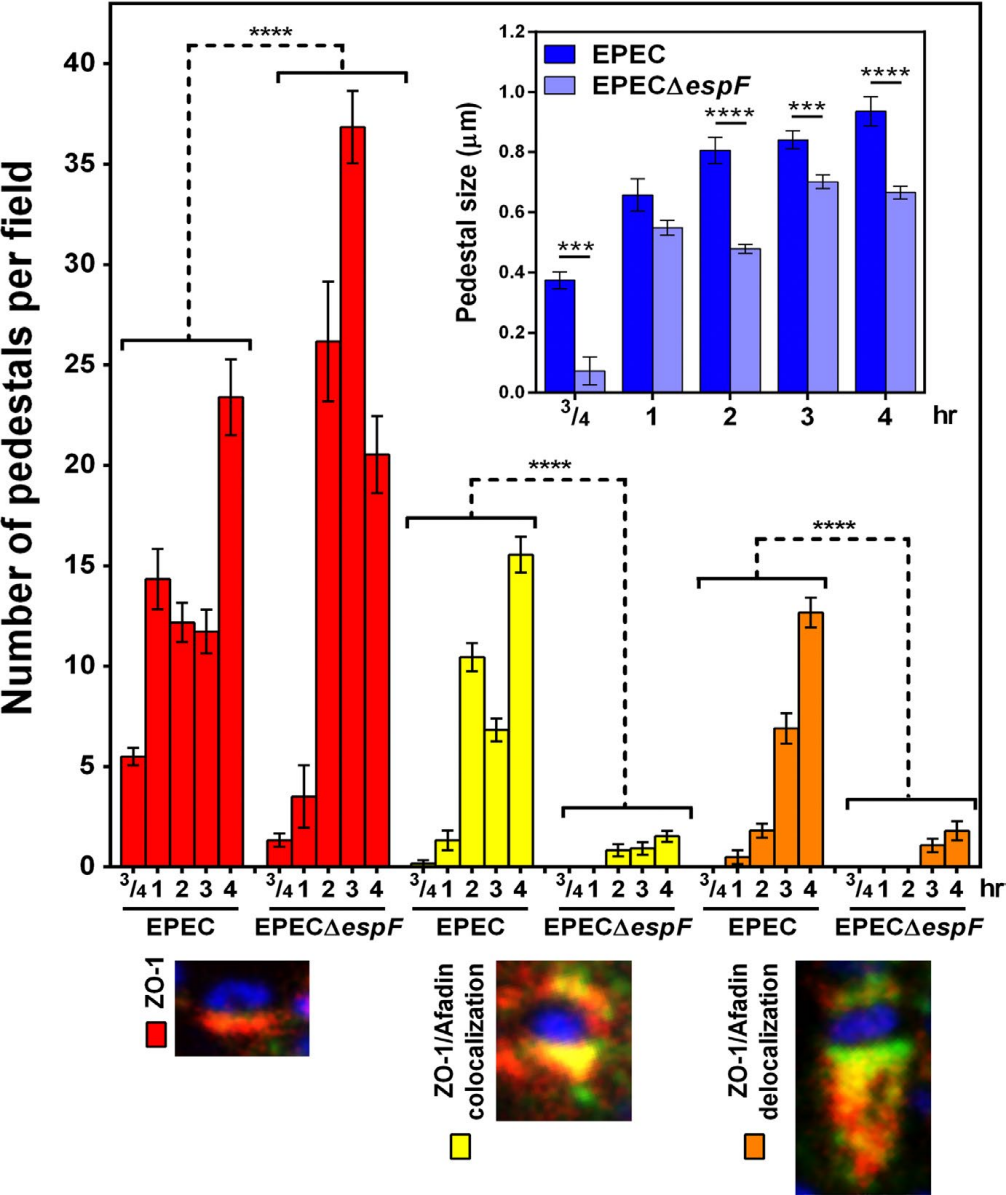
EspF induces the recruitment of ZO‐1 and afadin into the pedestals where their transitory interaction increases the pedestal size. Confocal microscopy images from kinetics of infection of L cells with EPEC or EPECΔ*espF* at a MOI of 0.5 were further analyzed by the Leica Lite software using images as those in from Figure 3 and S3. Dynamic of localization of ZO‐1 and afadin into the pedestal was analyzed by measuring the number of pedestals containing ZO‐1 (red bars), ZO‐1 and afadin colocalization (yellow bars), and ZO‐1 and afadin delocalization: separated in the stem and tip pattern, respectively (orange bars). A representative image of the pedestals with each of these characteristics is shown below the graphic; insert: kinetics of pedestal growth during the infection of L cells with EPEC or EPECΔ*espF*. The size of the pedestals was measured (µm) as mentioned before. Each infection time (EPEC and EPECΔ*espF*) was compared using one‐way ANOVA test, *n* = 3 independent experiments. ***p < .0005, ****p < .0001

In order to correlate pedestal size with these phases of maturation, we measured the size of pedestals formed by EPEC and EPECΔ*espF* during the infection kinetics (Figure 4 insert) and compared it with the pedestal number per cell and with pedestal maturation phases (ZO‐1 recruitment, colocalization of ZO‐1 and afadin, and delocalization of ZO‐1 and afadin) mentioned above (Figure 4 below). The pedestal size formed by EPEC (0.4 μm) at 45 min of infection progressively increased with the infection times, reaching a maximum at 4 hr (1 μm). In the case of pedestals formed by EPECΔ*espF* at 1 and 2 hr of infection (0.55 and 0.5 μm), these slightly increased in size at 3 hr of infection (0.65 μm) and they remained at the same size at 4 hr. Comparing the these pedestals’ size with the pedestal number in which only ZO‐1 signal was detected (phase 1), at 45 min and 1 hr of infection, the pedestals induced by EPEC were smaller in size and the number of these pedestals was higher (around 5 and 15 pedestals per field) than that observed in cells infected with EPECΔ*espF*; meanwhile, afadin was not recruited at these times (Figure 4). Conversely, the number of pedestals induced at 2 and 3 hr of EPEC infection containing only ZO‐1 was around two‐ to threefold lower than those formed by EPECΔ*espF*. These data correlated with a greater number of EPEC‐induced pedestals that displayed strong ZO‐1/afadin colocalization (phase 2), which was not detected in EPECΔ*espF*‐induced pedestals. Interestingly, we detected a population of pedestals that lacked ZO‐1/afadin colocalization at 3 hr of infection (phase 3). At 4 hr of infection, the number of pedestals per field containing only ZO‐1 increased to similar levels in both EPEC‐ and EPECΔ*espF*‐infected cells. However, in EPEC‐infected cells, pedestals exhibited ZO‐1/afadin colocalization (phase 2) as well as pedestals in which ZO‐1/afadin colocalization was lost (phase 3), and these phases were almost absent in EPECΔ*espF*‐infected cells (Figure 4).

**Figure 5:**
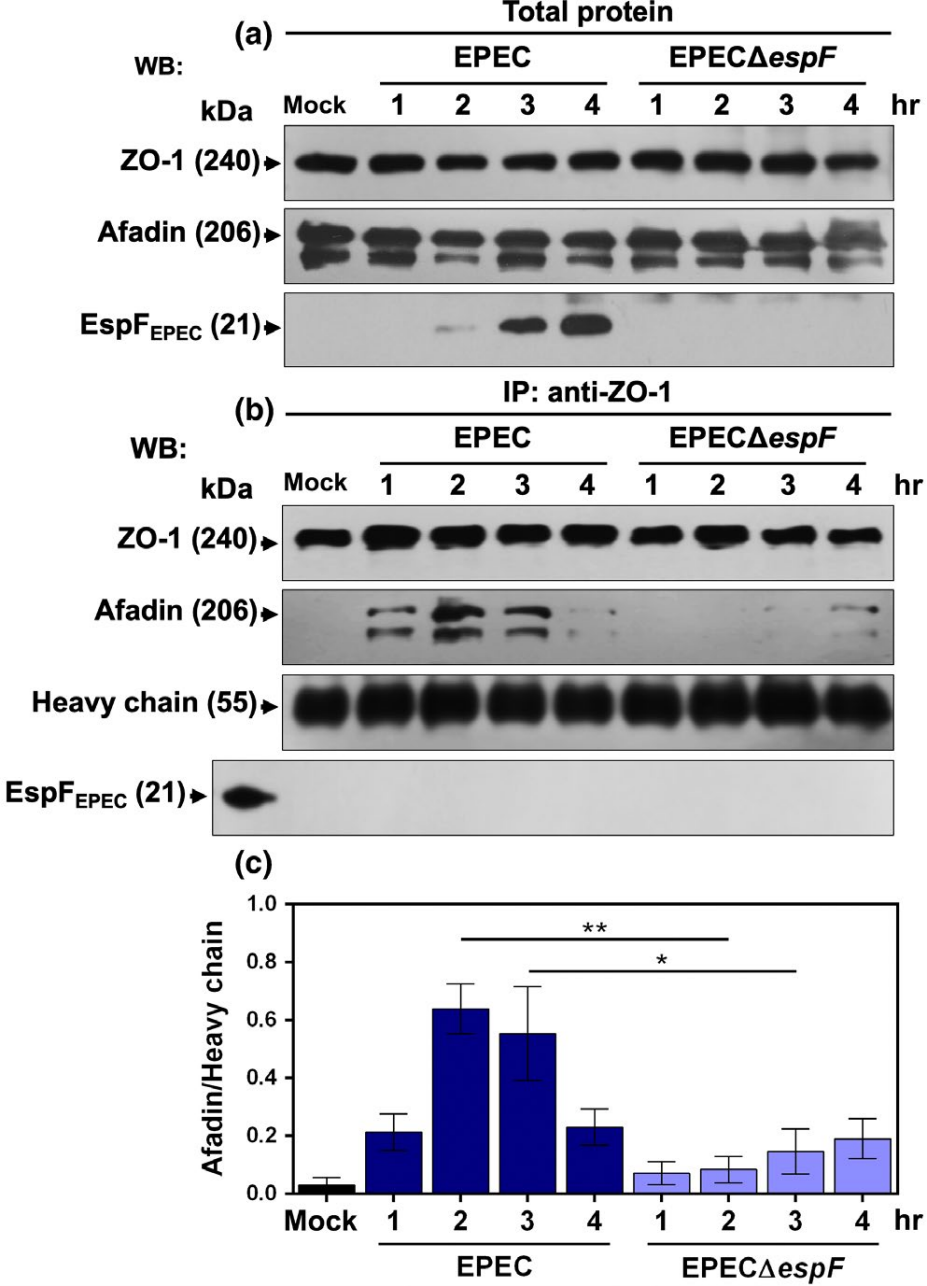
EspF is required for ZO‐1 and afadin interaction. L cells at 80% confluence were infected with EPEC and EPECΔ*espF* at a MOI of 5 for 1, 2, 3, and 4 hr. Infected cells were washed and lysed using RIPA buffer containing protease inhibitors (cOmplete^™^). Total proteins in the cell lysates were separated by SDS‐PAGE (a) or used for co‐immunoprecipitation assays using rabbit anti‐ZO‐1 antibodies (1 µg) and protein A‐agarose; the immunocomplex was separated by SDS‐PAGE (b). Both gels were transferred to PVDF membranes for analyzing by Western blot using antibodies against ZO‐1, afadin, and EspF. (c) Densitometric analyses of protein bands co‐immunoprecipitating with ZO‐1. Protein bands were analyzed using the Fiji 2.1.0 software, and data were plotted and analyzed using GraphPad Prism 6.0 software. Precipitated proteins were normalized using the heavy chain of the antibodies against ZO‐1 as protein load. All data were compared with mock cells using a one‐way ANOVA test and Tukey test, *n* = 3 independent experiments. *p < .05; **p < .005

We then sought to corroborate whether a physical ZO‐1/afadin interaction occurred in the pedestals and if EspF is participating in this process. L cells were mock‐infected or infected with either EPEC or EPECΔ*espF* for 1‐hr increments until 4 hr in total, and cell lysates were obtained to conduct immunoprecipitation assays using anti‐ZO‐1 antibodies. Immunocomplexes were analyzed by Western blot using anti‐ZO‐1, anti‐afadin, and anti‐EspF antibodies. In total lysates, total concentration of ZO‐1 and afadin did not change throughout the infection kinetics, whereas a time‐dependent increase in the amount of EspF was observed during wild‐type EPEC infection. Accordingly, EspF was not detected at any time point of infection with the mutant EPECΔ*espF* (Figure 5a). Anti‐ZO‐1 immunoprecipitated fractions were analyzed with anti‐afadin antibodies and revealed the presence of a concomitant ZO‐1/afadin complex at 1 hr of infection by EPEC. This interaction peaked at 2 and 3 hr of infection but was faintly detectable at 4 hr; meanwhile, EspF was not co‐immunoprecipitated with ZO‐1 nor as part of the ZO‐1/afadin complex. In contrast, the same analysis failed to detect any ZO‐1/afadin complexes in cell lysates from EPECΔ*espF*‐infected cells at all infection time points except at 4 hr, at which a faint signal of afadin was observed. As expected, no EspF‐immunoreactive band was detected in anti‐ZO‐1 immunocomplexes at any infection time point (Figure 5b), except in the control sample containing purified EspF. Densitometric analyses of three independent experiments showed that afadin co‐immunoprecipitated with ZO‐1 from 1 hr of infection with maximum interaction peaks at 2 and 3 hr of infection, and this interaction pattern was not detected in the absence of EspF (Figure 5c). These data support the results obtained in the colocalization analysis (see Figure 3i–l), in which the pedestals formed by EPEC had higher ZO‐1/afadin colocalization at 2 hr of infection, followed by a lack of colocalization at 4 hr of infection (see Figure 3q‐T). This lack of colocalization was the result of ZO‐1 and afadin segregation in the stem and tip pattern inside the pedestals, respectively. Although it is evident that EspF is required for this interaction, surprisingly EspF was not detected in the immunocomplexes, despite being found in the total lysates, which indicates that EspF is involved in promoting the interaction between ZO‐1 and afadin in a yet unknown mechanism.

### 2.4 ZO‐1 or afadin knockdown affects the pedestal growth induced by EPEC

**Figure 6:**
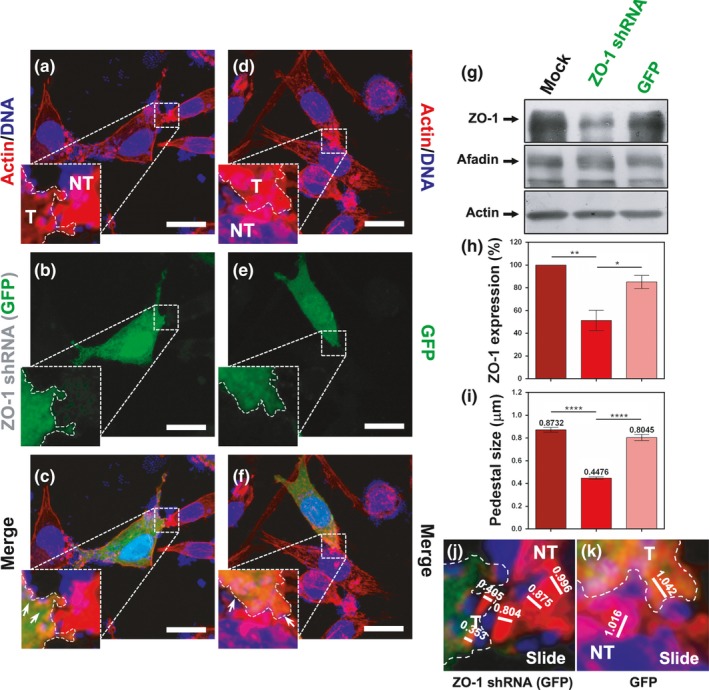
ZO‐1 knockdown induces a decrease in pedestal size induced by EPEC. (a–f) Role of ZO‐1 on the pedestal size. Knockdown (a–c) and normal (d–f) cells were fixed, permeabilized, and stained with DAPI (DNA, blue) and FAS (F‐actin, red). Transfected cells (T) expressing GFP (green) were delineated to separate them from nontransfected cells (NT); note the difference in pedestal size (arrows). Slides were analyzed by confocal microscopy, and each panel is projecting a zoom for showing actin pedestals. Bar: 20 µm. Sections of 0.5 μm were used to measure individual pedestals, 150 from three independent experiments (i.e., [j] and [k] are sections from panels [c] and [f], respectively). (g, h) ZO‐1 knockdown. L cells were transfected using pLL5.0 mZO1‐1 shRNA (Addgene plasmid #37215) [ZO‐1 shRNA (GFP)] or the empty pLL5.0 (GFP). Mock cells were treated only with Lipofectamine. Proteins from cell lysates were separated by SDS‐PAGE and analyzed by Western blot using antibodies against ZO‐1, afadin, and actin (g). Densitometric analyses of ZO‐1 expression (h) were performed with the protein bands using Fiji 2.1.0 software. ZO‐1 detection was normalized using actin bands. (i) Pedestals induced by transfected cells infected with EPEC were measured (µm) as mentioned before. Data were compared (*p < .05; **p < .005; ****p < .0001) using a one‐way ANOVA test, *n* = 3 independent experiments

**Figure 7:**
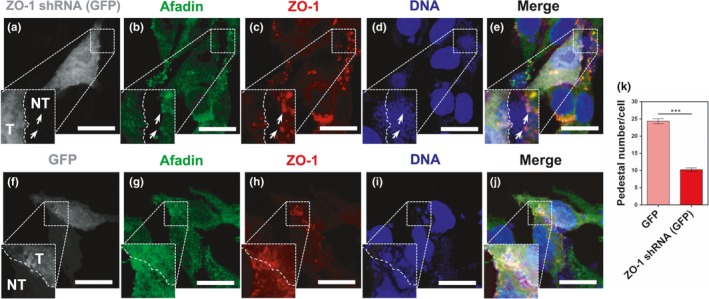
ZO‐1 knockdown in L cells decreases afadin recruitment to pedestals and pedestal number induced by EPEC. L cells were transfected using pLL5.0 mZO1‐1 shRNA (Addgene plasmid #37215) [ZO‐1 shRNA (GFP)] (a–e) or the empty pLL5.0 (GFP) (f–j). After 48 hr, cells were infected with EPEC for 4 hr. Infected cells were fixed, permeabilized, and stained with DAPI (DNA, blue). The stained cells were immunostained with a rabbit anti‐afadin polyclonal antibody followed by a biotin‐SP‐conjugated AffiniPure goat anti‐rabbit IgG and then rhodamine‐conjugated streptavidin (pseudocolored green), and with a mouse anti‐ZO‐1 monoclonal antibody followed by a secondary antibody, CY5‐donkey anti‐mouse IgG (pseudocolored red). Transfected cells were detected by GFP expression (pseudocolored gray) and were delineated to separate them from nontransfected cells (NT); note the difference in pedestal size and number (arrows) in transfected cells. Slides were analyzed by confocal microscopy, and each panel is projecting a zoom for showing actin pedestals beneath adhered bacteria. Bar: 20 µm. (k) The number of actin pedestals per cells was quantified in 30 cells per experiment in three independent experiments using the Leica Lite software, *n* = 3. ***p < .0005, using a one‐way ANOVA test

**Figure A4:**
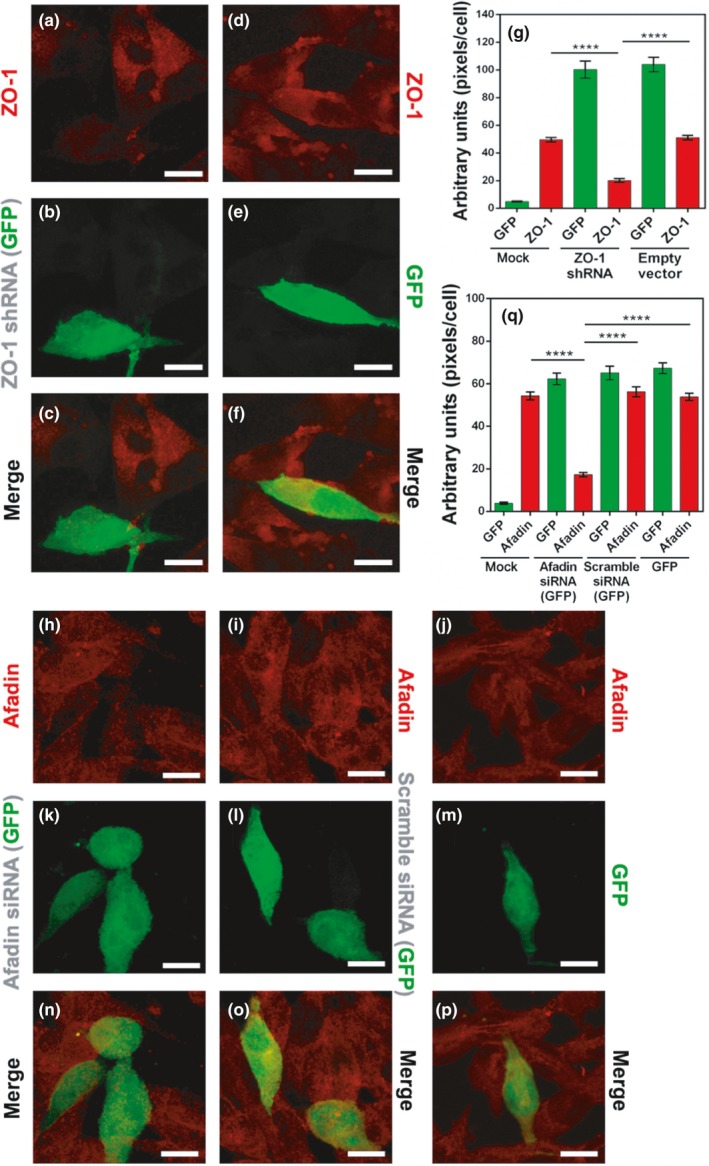
ZO‐1 and afadin knockdown in L cells. For ZO‐1, L cells were transfected using pLL5.0 mZO1‐1 shRNA (Addgene plasmid #37215) [ZO‐1 shRNA (GFP)] (a–c) or the empty pLL5.0 (GFP) (d–f). For afadin, L cells were transfected using synthetic afadin siRNA [Afadin siRNA (GFP)] (h–j) or a scramble siRNA [Scramble siRNA (GFP)] (k–m) or the pGreen Lantern‐1 (GFP) alone (n–p). Mock cells were treated only with Lipofectamine. After 48 hr, cells were fixed, permeabilized, and immunostained with a mouse anti‐ZO‐1 monoclonal antibody or rabbit anti‐afadin polyclonal antibody followed by a secondary antibody, CY5‐donkey anti‐mouse IgG (pseudocolored red) or a secondary antibody, CY5‐donkey anti‐rabbit IgG (pseudocolored red). Transfected cells were detected by GFP expression. Slides were analyzed and recorded by confocal microscopy (63× zoom 3). Bar: 20 µm. (g, q) Red (ZO‐1 or afadin) or green (GFP) pixels into 20 cells in each transfected condition were measured using the Fiji 2.1.0 software (ImageJ), *n* = 3. ***p < .0005, using one‐way ANOVA test

In order to corroborate the role of ZO‐1 and afadin on the pedestal maturation, we opted for a silencing approach of either of the proteins using two commercial previously tested knockdown methods: a plasmid expressing a shRNA for ZO‐1 and a siRNA for afadin (Fanning Lab deposited in Addgene) (Yamamoto et al., 2015). Thus, ZO‐1 silencing assays were achieved following the transfection of L cells with a plasmid expressing a shRNA (ZO‐1 shRNA) specific to silence ZO‐1 in mouse. Lysates from ZO‐1 knockdown L cells were analyzed by Western blot and densitometry. In cells transfected with ZO‐1 shRNA, ZO‐1 expression was reduced to about 50% in comparison with those transfected with the empty vector (GFP) and cells treated only with lipofectin (mock) (Figure 6g, h). Additionally, transfected knockdown cells were also confirmed as ZO‐1‐depleted cells detected by immunofluorescence using anti‐ZO‐1 antibodies (Figure A4a–g; and see Figure 7c, h). Cells under these conditions were infected with wild‐type EPEC for 4 hr, and the pedestal size (see Figure 6j, k) was measured using confocal microscopy (Figure 6i). Pedestals formed in ZO‐1 knockdown (T) cells were significantly smaller (0.44 μm) than those in nontransfected cells (NT) (0.87 μm) in the same field of view (Figure 6a, b, and c), whereas in cells transfected with the empty vector (T), the pedestals (0.8 μm) were similar to those in nontransfected cells (NT) (0.87 μm) in the same field of view (Figure 6d, e, and f). Thus, ZO‐1 knockdown correlated with the pedestal growth by reducing the size of pedestals at half of the normal size in wild‐type EPEC (Figure 6i).

To correlate pedestal size with afadin recruitment to the actin‐pedestal structure in ZO‐1 knockdown L cells, ZO‐1 shRNA‐transfected cells and the cells transfected with the empty vector (GFP) were infected with EPEC for 4 hr. Cells under these different conditions were fixed, permeabilized, and stained using anti‐ZO‐1 and anti‐afadin antibodies. As expected, in each optical field we detected a population of nontransfected cells (NT) and another of transfected cells (T), detected by GFP expression. Thus, in ZO‐1 shRNA‐transfected cells (T), the afadin signal was practically undetectable into the pedestals (Figure 7a–e) and only small traces of ZO‐1 signal were visualized, contrasting with the neighboring NT cells with which they are making cell–cell contact. In these NT cells, the detection and colocalization of ZO‐1 and afadin were evident, whereas in both control cells, transfected with GFP (T), and neighboring NT cells, the colocalization of ZO‐1 and afadin into the pedestals was detected (Figure 7f–j). Moreover, the lack of ZO‐1 in the knockdown cells drastically decreased the number of pedestals per cell in comparison with the cells transfected with the empty vector (Figure 7k), indicating the relevance of ZO‐1 recruitment to the pedestals during the infection by EPEC.

**Figure 8:**
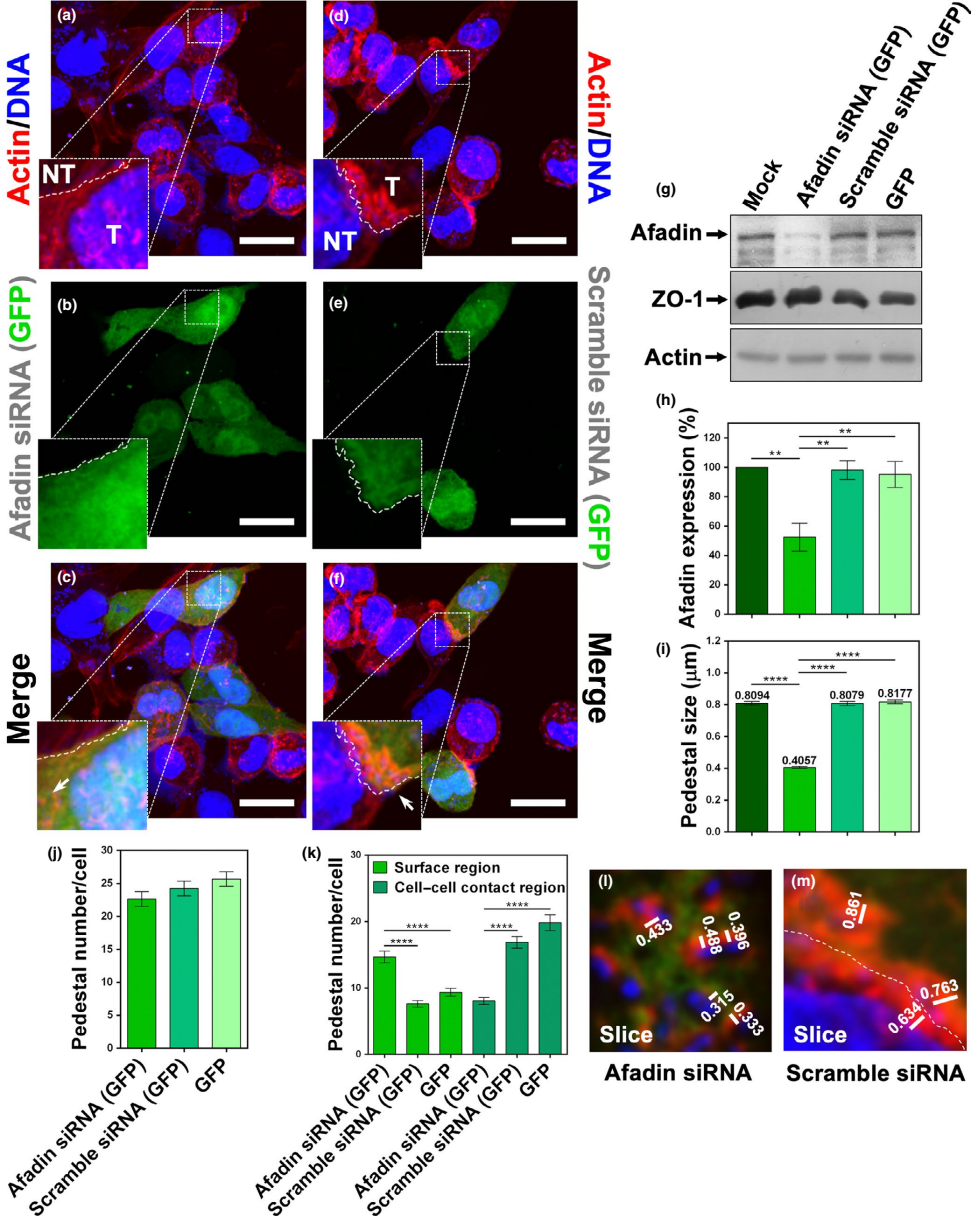
Afadin knockdown induces a decrease in pedestal size and produces pedestal redistribution along the cells. (a–f) Role of afadin on the pedestal size. Knockdown (a–c) and normal (d–f) cells were fixed, permeabilized, and stained with DAPI (DNA, blue) and FAS (F‐actin, red). Transfected cells (T) expressing GFP (green) were delineated to separate them from nontransfected cells (NT); note the difference in pedestal size (arrows). Slides were analyzed by confocal microscopy, and each panel is projecting a zoom for showing actin pedestals. Bar: 20 µm. Sections of 0.5 μm were used to measure pedestals, 150 from three independent experiments (i.e., L and M are sections from panels [c] and [f], respectively). (g, h) Afadin knockdown. L cells were transfected using synthetic afadin siRNA or a scramble siRNA and pGreen Lantern‐1 (GFP) [Afadin siRNA (GFP) and Scramble siRNA (GFP), respectively] or the pGreen Lantern‐1 (GFP) alone. Proteins from cell lysates were analyzed by Western blot using antibodies against afadin, ZO‐1, and actin (g). Densitometric analyses of afadin expression (h) were performed with the protein bands using Fiji 2.1.0 software (*n* = 3). Afadin detection was normalized using actin bands. (i) Pedestals induced by transfected cells infected with EPEC were measured (µm) as mentioned before. (j) Number of pedestals per cell. The actin pedestals were quantified in 30 cells from three independent experiments using the Leica Lite software. (k) Distribution of pedestals into the cell. The number of pedestals per cell formed either in cell–cell contacts or in surface regions (no cell–cell contact) was quantified in 30 cells in three independent experiments using the Leica Lite software. Data were compared (**p < .005; ****p < .0001) using a one‐way ANOVA test, *n* = 3 or 4

Due to the role of ZO‐1 in the recruitment of afadin to pedestals, in the pedestal maturation, and in the increase of pedestal number formed by EPEC, we decided to determine the role of afadin in these phenotypes. To do that, L cells were transfected with synthetic siRNA specific to silence afadin of mouse (afadin siRNA), as well as with unspecific siRNA as a negative control (scramble siRNA). Both siRNAs were cotransfected with a plasmid expressing GFP as a marker of transfection. Knockdown of afadin was detected in cell lysates by Western blot and analyzed by densitometry. In cells transfected with afadin siRNA, the signal of this protein decreased around 50% in comparison with those cells transfected with the scramble siRNA or only GFP, or cells treated only with Lipofectamine (mock) (Figure 8g, h). These knockdown cells were also confirmed by immunofluorescence using anti‐afadin antibodies, and the different cell populations containing or lacking afadin were quantified in cells treated with afadin siRNA, scramble siRNA, or GFP alone (Figure A4h–q). The L cells with the different transfection schemes were infected with EPEC for 4 hr. In afadin siRNA‐transfected cells, the pedestals formed were 50% smaller (Figure 8a–c, i) than those formed in scramble siRNA‐transfected cells (Figure 8d–f, i), which were similar to those in GFP‐transfected cells or mock cells (Figure 8i). Interestingly, unlike ZO‐1 knockdown, afadin knockdown did not decrease the number of pedestals per cell, and they were like those in scramble siRNA‐transfected cells or in GFP‐transfected cells (Figure 8j). These data indicate that afadin is key for pedestal maturation (see transfected cells in 8a vs. 8d), but it is not crucial in controlling the pedestal number per cell formed by EPEC. Moreover, a careful analysis of the confocal microscopy images showed that afadin knockdown caused a different distribution of pedestals along the infected cells. By counting pedestals on the cell surface region and on the cell–cell contact region during the different treatments, there was a twofold increase in the number of EPEC‐induced pedestals at the cell surface region of afadin knockdown cells compared to the cell–cell contact region (Figure 8k). Conversely, in cells treated only with Lipofectamine (mock) or transfected with scramble siRNA or only with GFP, most pedestals were found on the cell–cell contact region.

These data indicate that ZO‐1 is essential for pedestal maturation and that its recruitment to the pedestal allows afadin recruitment to start a transient interaction leading to pedestal growth in enriched ZO‐1 sites. These sites support the segregation of ZO‐1 and afadin into the stem and tip of pedestals, respectively, to finalize the pedestal growth. Interestingly, data from the afadin knockdown cells suggest that afadin deficiency impedes the formation of ZO‐1‐rich sites at cell–cell contacts and that the relationship between ZO‐1/afadin interaction dynamics and pedestal maturation is more complex.

### 2.5 Models of polarized cells forming stable intercellular junction also reproduce the phases for pedestal maturation

**Figure A5:**
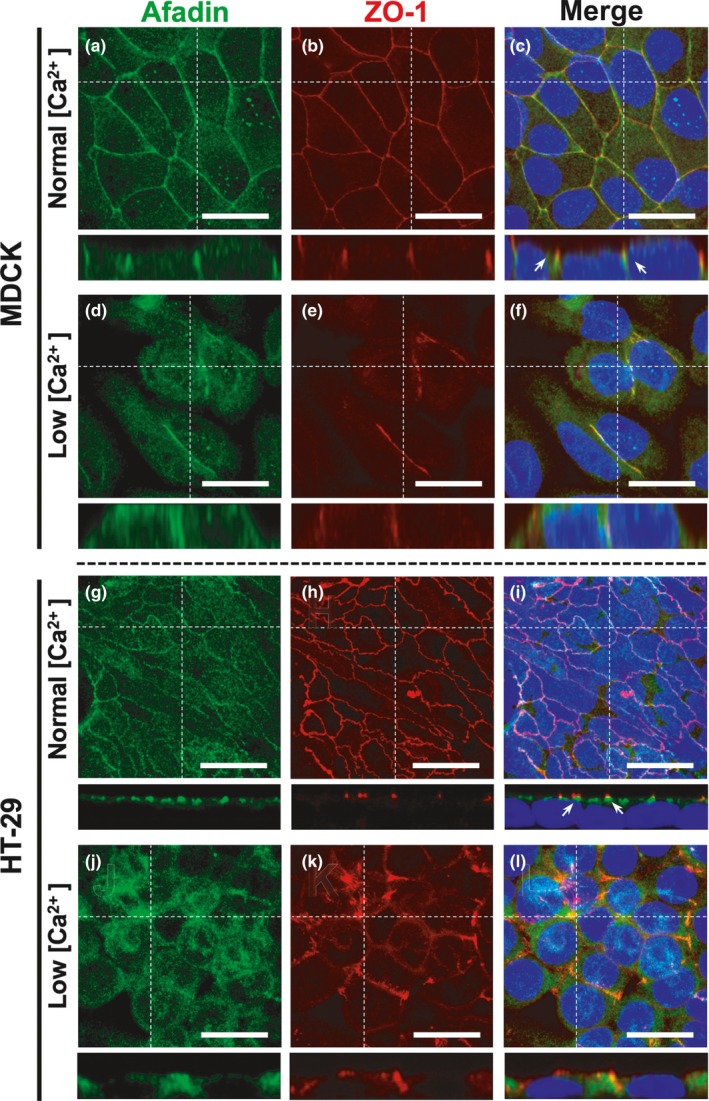
The calcium switch model in MDCK and HT‐29 cells for analyzing assembly–disassembly of afadin and ZO‐1 from intercellular junctions. MDCK and HT‐29 cells were seeded at 100% of confluency for 5 and 10 days, respectively, and then subjected to calcium switch assay: MDCK (a–c) and HT‐29 (g–i) cells were kept in DMEM containing normal concentration of calcium, without antibiotics and bovine fetal serum (BFS) for 6 hr. MDCK (d–f) and HT‐29 (j–l) cells were washed and kept in fresh DMEM containing low concentration of calcium, without antibiotics and bovine fetal serum (BFS) for 6 hr. Cells under the different conditions were fixed, permeabilized, and stained with DAPI to detect nuclear DNA (blue). The stained cells were immunostained with a rabbit anti‐afadin polyclonal antibody followed by a biotin‐SP‐conjugated AffiniPure goat anti‐rabbit IgG and then fluorescein‐conjugated streptavidin (green), and a mouse anti‐ZO‐1 monoclonal antibody followed by a secondary antibody, CY5‐donkey anti‐mouse IgG (pseudocolored red). Slides were analyzed and recorded by confocal microscopy (63× zoom 1). Each panel shows maximum projection and a *z*‐section (*zx*). Bar: 20 µm. Arrows point out the classical localization of ZO‐1 and afadin in the intercellular junctions

To probe the ZO‐1 and afadin dynamics in polarized cells with high and low transepithelial electrical resistances (Dukes, Whitley, & Chalmers, 2011; Gagnon, Zihler Berner, Chervet, Chassard, & Lacroix, 2013), we once again used the calcium switch model in MDCK cells and an intestinal cell line, HT‐29. First, the distribution pattern of afadin was monitored in HT‐29 and MDCK cells that were maintained in either normal‐ or low‐calcium conditions during 6 hr. The cells were fixed, permeabilized, and stained using anti‐afadin and anti‐ZO‐1 antibodies. Samples were analyzed by confocal microscopy. In the case of MDCK cells (cultured by 5 day) at normal‐calcium condition, afadin and ZO‐1 were localized in a continuous pattern in the cell periphery, in the classical chicken wire pattern, without displaying a robust colocalization (Figure A5a–c). In fact, in a *z*‐section of the intracellular junction it was possible to detect the classical localization of ZO‐1 and afadin: ZO‐1 at the apical junction (in the TJ) and afadin below ZO‐1 (in the AJ) (Figure A5c, arrows). When the calcium concentration was low, the continuous pattern of afadin and ZO‐1 in the cell periphery was lost; they were localized only in the cytoplasm or in residual regions of the cell–cell contact, where these two proteins were discretely colocalized in some sites (Figure A5d–f). These results were reproduced in the intestinal cell line of a low transepithelial electrical resistance, where HT‐29 cells (cultured by 10 day) were clearly polarized showing the classical localization of ZO‐1 apically in the TJs and afadin immediately below in the AJs in normal‐calcium conditions (Figure A5g–i), whereas in low calcium concentration, the TJs and AJs were disassembled and ZO‐1 and afadin were delocalized in the cytoplasm (Figure 5A j–l).

**Figure 9:**
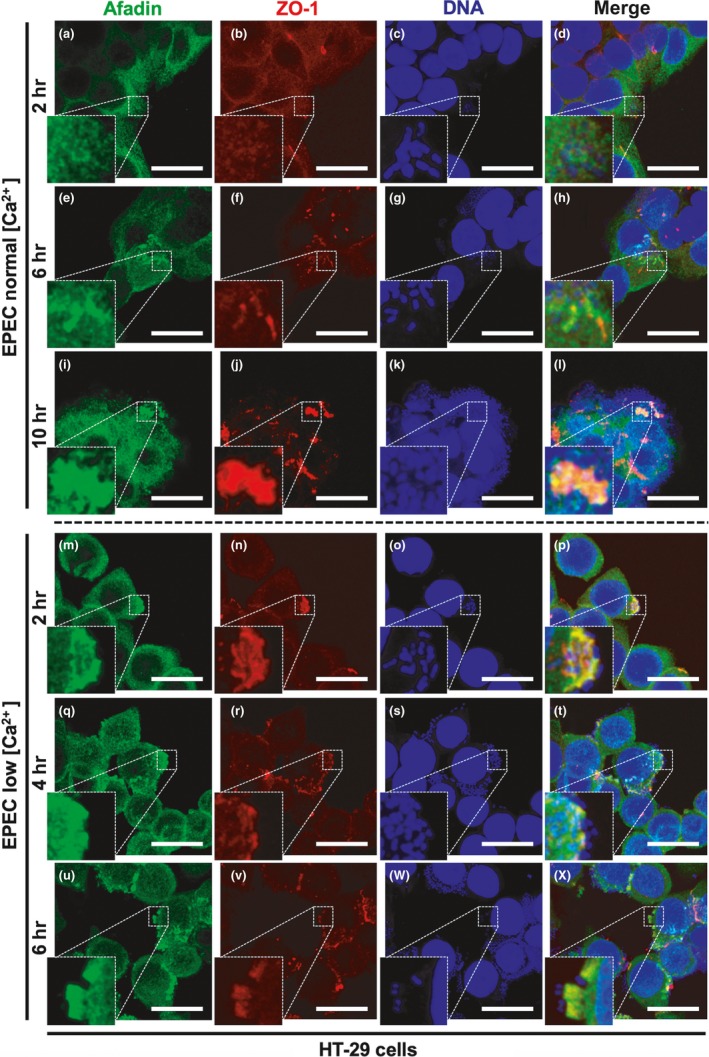
Intestinal epithelial cells reproduce all phenotypes detected in L and MDCK cell models: ZO‐1 and afadin recruitment for pedestal maturation. Human epithelial cells, HT‐29, were treated as MDCK in the calcium switch model. HT‐29 cells under normal calcium concentration or low calcium concentration were infected with EPEC for 2, 6, and 10 hr or 2, 4, and 6 hr, respectively. After infection times, cells under the different conditions were fixed, permeabilized, and stained with DAPI (DNA, blue). The stained cells were immunostained with a rabbit anti‐afadin polyclonal antibody followed by a biotin‐SP‐conjugated AffiniPure goat anti‐rabbit IgG and then fluorescein‐conjugated streptavidin (green), and a mouse anti‐ZO‐1 monoclonal antibody followed by a secondary antibody, CY5‐donkey anti‐mouse IgG (pseudocolored red). Slides were analyzed and recorded by confocal microscopy (63× zoom 3). Each panel is projecting a zoom for showing actin pedestals beneath adhered bacteria. Bar: 20 µm

To trace the afadin redistribution during the infection by EPEC in HT‐29 cells under normal‐ or low‐calcium conditions, the HT‐29 cells were infected by EPEC for 2, 6, and 10 hr or 2, 4, and 6 hr, respectively. In normal‐calcium conditions and at 2 hr of infection, afadin and ZO‐1 were excluded from intercellular junctions, but only ZO‐1 was incipiently detected in the actin pedestals beneath adhered bacteria (Figure 9a–d). At 6 hr of infection (Figure 9e–h), ZO‐1 and afadin were localized into the pedestals, without colocalization, with the impression that ZO‐1 mark was in the center of the pedestal structure and afadin was externally coating ZO‐1 in the core. At 10 hr of infection (Figure 9i–l), ZO‐1 and afadin were clearly colocalized in most pedestals. On the other hand, this whole process was expedited in low‐calcium condition, since at 2 hr of infection (Figure 9m–p), EPEC was able to recruit ZO‐1 and afadin into the pedestals, where these two proteins strongly colocalized. At 4 hr of infection (Figure 9q–t), ZO‐1 and afadin were delocalized into the pedestals, and in some pedestals, these proteins were already separated in the stem and tip pattern, respectively. At 6 hr of infection (Figure 9u–x), the stem and tip distribution for both proteins was in process in most pedestals, which were evidently bigger.

**Figure A6:**
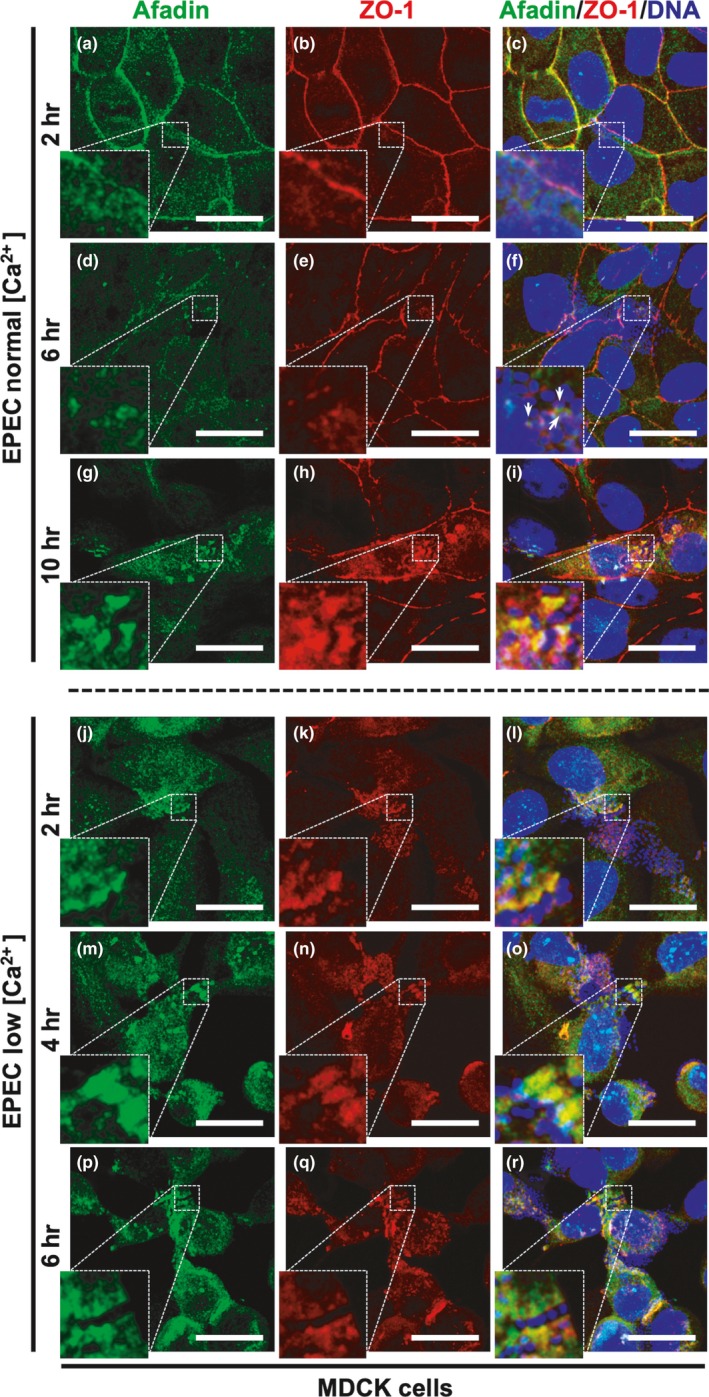
Afadin is disassembled before ZO‐1, but it is recruited to the pedestals after ZO‐1 and low [Ca^2+^] facilitates this process. (a–i) MDCK cells under normal concentration of calcium, without antibiotics and bovine fetal serum (BFS). (j–r) MDCK cells under low concentration of calcium, without antibiotics and bovine fetal serum (BFS). Cells under normal calcium concentration or low calcium concentration were infected with EPEC for 2, 6, and 10 hr or 2, 4, and 6 hr, respectively. After infection times, cells under the different conditions were fixed, permeabilized, and stained with DAPI to detect nuclear DNA (blue). The stained cells were immunostained with a rabbit anti‐afadin polyclonal antibody followed by a biotin‐SP‐conjugated AffiniPure goat anti‐rabbit IgG and then fluorescein‐conjugated streptavidin (green), and a mouse anti‐ZO‐1 monoclonal antibody followed by a secondary antibody, CY5‐donkey anti‐mouse IgG (pseudocolored red). Slides were analyzed and recorded by confocal microscopy (63× zoom 3). Each panel is projecting a zoom for showing actin pedestals beneath adhered bacteria. Bar: 20 µm

Remarkably, in the classical calcium switch model in MDCK cells all the processes described above were replicated but at a slight low velocity in the pedestal maturation, mainly in the process of ZO‐1 and afadin disassembly (Figure A6). In normal‐calcium conditions and at 2 hr of infection, afadin was partially excluded from the intercellular junctions, whereas ZO‐1 was maintained in the intercellular junctions, which appeared to be under stress, but none of these two proteins was detected in the actin pedestals at the bacteria attachment sites (Figure A6a–c). However, at 6 hr of infection (Figure A6d–f), afadin was progressively excluded from the intercellular junctions, while ZO‐1 started to be detected as a discontinuous labeling (right beneath the adhered bacteria) and highly stressed in the intracellular junctions. Both proteins began to relocate incipiently in the actin pedestals without colocalization. At 10 hr of infection and in normal‐calcium condition, ZO‐1 and afadin colocalized inside of the pedestal structure, mainly in the biggest pedestals. In contrast, at low calcium concentration at 2 hr of infection (Figure A6j–l), EPEC was able to recruit ZO‐1 and afadin to the pedestal structures, where both proteins were strongly colocalized. At 4 hr of infection (Figure A6m–o), ZO‐1 and afadin colocalized to a greater extent in the pedestals; in addition, the initiation of the delocalization of afadin and ZO‐1 was detected, and in some pedestals, these proteins were already separated in the stem and tip pattern, respectively, as previously mentioned. At 6 hr of infection (Figure A6p–r), stem and tip distribution of ZO‐1 and afadin, respectively, was more evident in many pedestals, although in some ones these two proteins were still colocalized.

All these data indicate that in high‐ or low‐electrical‐resistance epithelia such as MDCK and HT‐29 cells, EPEC can recruit ZO‐1 and afadin into the pedestals at late times of infection in normal‐calcium conditions and this process is streamlined at low calcium concentration. Even though afadin is disassembled before ZO‐1, it is necessary that ZO‐1 be recruited to the pedestals so that afadin can be detected inside these structures. These data clearly indicate that the efficient recruitment of ZO‐1 in the pedestals requires the disassembly of proteins from intercellular junctions. Then, ZO‐1 recruits afadin to colocalize in the pedestal and a transient interaction between these two proteins begins, leading to their distribution in stem and tip in the pedestal structure, respectively, that culminates in the pedestal maturation.

**Figure 10:**
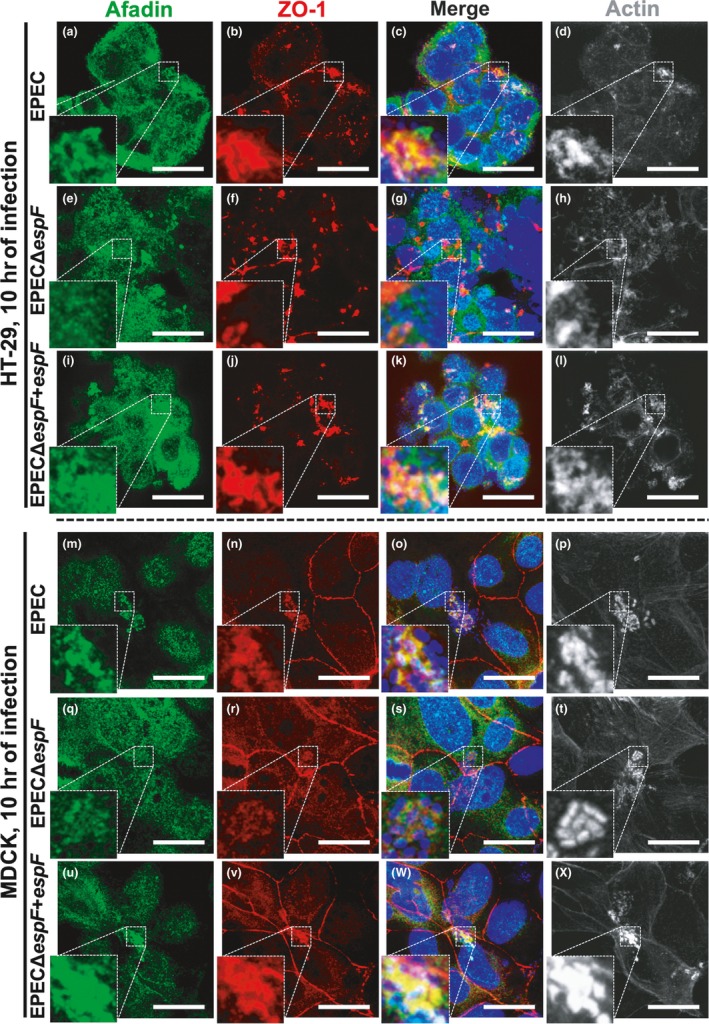
ZO‐1 and afadin colocalization is induced by EspF in intestinal epithelial cells (HT‐29) and in the classical polarized MDCK cells. (a–l) HT‐29 cells were infected with EPEC (a–d), EPECΔ*espF* (e–h), or EPECΔ*espF‐pespF* (i–l) for 10 hr. (m–x) MDCK cells were infected with EPEC (M‐P), EPECΔ*espF* (Q‐T), or EPECΔ*espF‐pespF* (u–x) for 10 hr. Cells were fixed, permeabilized, and stained with DAPI (DNA, blue) (c, g, k, o, s, and w) and FAS (F‐actin, pseudocolored gray) (d, h, l, p, t, and x). The stained cells were immunostained with a mouse anti‐ZO‐1 monoclonal antibody followed by a secondary antibody, CY5‐donkey anti‐mouse IgG (pseudocolored red) (b, f, j, n, r, and v), and with a rabbit anti‐afadin polyclonal antibody followed by a biotin‐SP‐conjugated AffiniPure goat anti‐rabbit IgG and then fluorescein‐conjugated streptavidin (green) (a, e, i, m, q, and u). Slides were analyzed and recorded by confocal microscopy (63X zoom 3). Bar: 20 µm

To clearly show the role of EspF in this maturation process in polarized cells such intestinal cell lines or the classical polarized model, MDCK cells, both cell lines were infected by EPEC, the *espF* mutant (EPECΔ*espF*), and the mutant complemented by *espF* (EPECΔ*espF‐pespF)* for 10 hr. As expected, both HT29 (Figure 10a–d) and MDCK (Figure 10m–p) infected with the EPEC wild type showed colocalization between ZO‐1 and afadin inside the pedestal structures. Remarkably, the infection of both HT‐29 (Figure 10e–h) and MDCK (Figure 10q–t) cells with EPECΔ*espF* did not cause this colocalization of ZO‐1 and afadin into the pedestal structures and these pedestals were visibly smaller than those induced by EPEC wild type (i.e., Figure 10p vs. Figure 10t). However, when the mutant was complemented (EPECΔ*espF‐pespF)*, both infected HT‐29 (Figure 10i–l) and MDCK (Figure 10u–x) cells showed ZO‐1 and afadin colocalization into the pedestal structures and again these pedestals were clearly bigger than those produced by EPEC wild type (Figure 10p vs. x) due to *espF* expression *in trans*, as we previously showed during the infection by REPEC (Peralta‐Ramirez et al., 2008).

**Figure 11:**
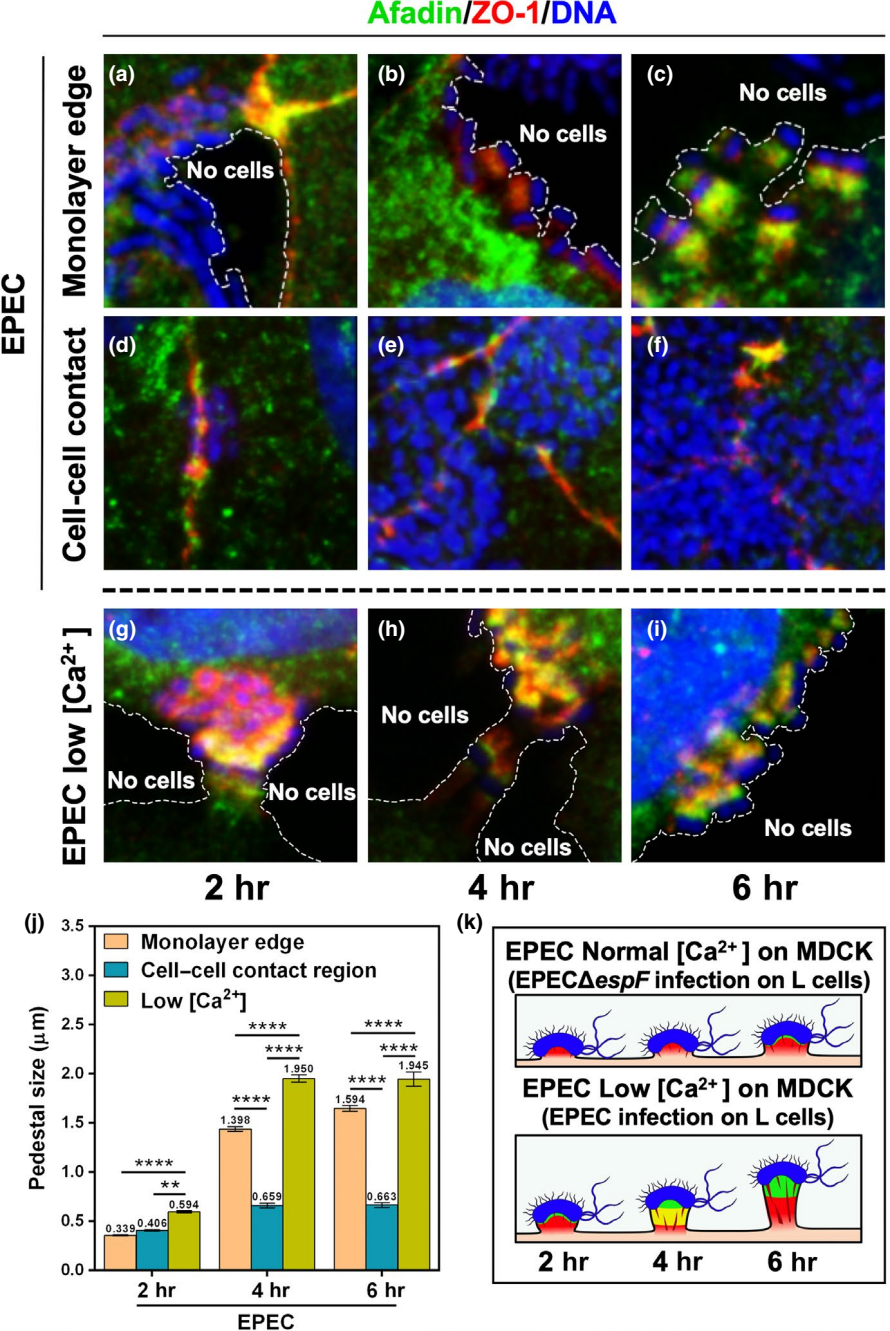
Disassembled ZO‐1 and afadin induced by EspF, low [Ca^2+^] or in the monolayer edges, allow sequential recruitment of both proteins to the pedestal for their maturation. (a–f) MDCK cells in DMEM containing normal concentration of calcium. (g–i) MDCK cells in fresh DMEM containing low concentration of calcium. Cells were infected with EPEC for 2, 4, and 6 hr. The cells were fixed, permeabilized, and stained with DAPI (DNA, blue). The stained cells were immunostained with a rabbit anti‐afadin polyclonal antibody followed by a biotin‐SP‐conjugated AffiniPure goat anti‐rabbit IgG and then fluorescein‐conjugated streptavidin (green), and a mouse anti‐ZO‐1 monoclonal antibody followed by a secondary antibody, CY5‐donkey anti‐mouse IgG (pseudocolored red). Slides were analyzed by confocal microscopy as mentioned before. Bar: 20 µm. Dotted lines delimit the cell edges (absence of neighboring cells). (j) Pedestals were measured (µm) using the Leica Lite software. Data were compared (*p < .05; ****p < .0001) using a one‐way ANOVA test, *n* = 3 independent experiments. (k) Schematic representation of the similarities between pedestal formed by EPEC in MDCK cells on normal [Ca^2+^] medium or formed by EPEC∆*espF* in L cells (top), and pedestals formed by EPEC in MDCK cells on low [Ca^2+^] medium or formed by EPEC in L cells (below)

To corroborate that ZO‐1 and afadin availability (disassembled from intercellular junctions) allows the pedestal maturation induced by EspF, we decided to explore those pedestals formed at the edge of MDCK cell monolayers, where the cells were migrating and the intercellular junctions have not yet closed, so that the proteins that constitute them were readily available in the cytoplasm. At 2 hr of EPEC‐induced infection, the pedestals that were formed at the edge of the monolayer had already initiated ZO‐1 recruitment (Figure 11a), while those formed at the cell–cell contacts were erected at sites where ZO‐1 is assembled in the intercellular junctions, meanwhile displaying a similar pedestal size (0.4 μm) for both populations (Figure 11d). At 4 hr of infection, pedestals at the monolayer edge recruited a high amount of ZO‐1, and afadin recruitment was initiated but was not yet clearly detected into the pedestals (Figure 11b). In contrast, the pedestals formed on the intercellular junctions at 4 hr of infection did not recruit much of these two proteins (Figure 11e), and the pedestals were smaller (0.66 vs. 1.4 μm) than those produced at the edge of the monolayer (Figure 11j). At 6 hr of infection, both ZO‐1 and afadin were recruited in every pedestal formed at the edge of the monolayer with different maturation states: In some pedestals, there was colocalization; in other pedestals, both proteins were in the process of delocalization; and in lesser number, the stem and tip pattern was observed for ZO‐1 and afadin, respectively (Figure 11c). In addition, these latter pedestals were larger (1.6 μm) than those formed at 2 and 4 hr of infection (0.34 and 1.4 μm, respectively) at this edge region (Figure 11j). In contrast, the pedestals formed at the same time on the intercellular junctions showed very low signals of these two proteins (Figure 11f), and these pedestals remained small (0.6 μm) as were those formed at 2 and 4 hr in this same region (Figure 11j).

These data support the notion that the maturation process of actin pedestals depends on EspF and on the cytoplasmic availability of ZO‐1 and afadin, due to their disassembly from intracellular junctions. This mechanism involves first a ZO‐1/afadin interaction and second a separation of these proteins at both ends of the pedestals leading to the growth of these structures (Figure 11k). Indeed, the similar phenotype was observed in pedestals that were either present in the monolayer edges (see Figure 11c) or formed during the cell incubation at low calcium concentration (Figure 11i), a condition for which the processes previous to pedestal maturation were more evident (Figure 11g–h). In fact, these pedestals were slightly larger (1.9 vs. 1.5 μm) than those detected in the monolayer edges. This size difference was probably due to the higher availability of ZO‐1 and afadin in conditions created by the calcium switch, which made the cell–cell contacts unfavorable, hence the disassembly of intercellular junctions generating more edges than at a monolayer front.

## 3 DISCUSSION

EspF is a multifunctional protein that, once injected into epithelial cells, as an effector protein of the T3SS, is involved in subvert various cell processes in the cytoplasm as well as in some organelles (Holmes et al., 2010). EspF is associated with multiple functions, most of them into the cytosol of the host cell, several of which could be a consequence of intracellular junction disruption and/or cytoskeletal rearrangements (Ugalde‐Silva et al., 2016). Initially, it was reported that EspF was not involved in pedestal formation since, using an isogenic mutant, pedestals are formed in the absence of EspF (McNamara et al., 2001). Indeed, we found here that EspF is not involved in pedestal formation; however, this effector is relevant for pedestal growth by showing that an isogenic mutant forms smaller pedestals than those formed by wild‐type EPEC. Furthermore, small pedestals formed by the isogenic mutant are mainly located in intercellular junctions instead of along the cell as those formed by the wild‐type EPEC. In EPEC‐infected cells, these structures recruit ZO‐1, correlating with a fall in the transepithelial electrical resistance (Peralta‐Ramirez et al., 2008). Here, we showed that EspF is essential for pedestal maturation. EspF is required for ZO‐1 disassembly from the TJs, which leads to ZO‐1 recruitment into the pedestal structure. ZO‐1 recruitment is the first step for pedestal growth, where enriched zones of ZO‐1 are needed, which are present in cell–cell contacts, in already formed TJs, or in cells where these proteins are highly disassembled. ZO‐1 recruitment into pedestals is also required for the afadin recruitment to these structures, and thereby EspF will be also required. Afadin recruitment is needed for a transient interaction with ZO‐1 and, at the end of this interaction, afadin is recruited to the tip of the pedestal and ZO‐1 to the stem of the pedestal, and this separation leads to pedestal growth. Remarkably, in ZO‐1 knockdown cells, the pedestals were significantly smaller and the number of pedestals decreased, whereas in afadin knockdown cells, the pedestals were also smaller, but the number of pedestals was similar to normal cells. However, in these cells the pedestal distribution changed by favoring more pedestal formation along the cell surface than in cell–cell contacts.

An interesting finding by us and other groups is that EPEC expresses tropism toward intercellular junctions (Pedersen et al., 2017; Peralta‐Ramirez et al., 2008; Ugalde‐Silva et al., 2016). We found here that this tropism does not depend on EspF since both wild‐type EPEC and an isogenic *espF* mutant bind to the intercellular junctions (Figure 1). However, the *espF* mutant is unable to cause discontinuity of ZO‐1 along these intercellular junctions as the wild‐type EPEC does. Both strains form pedestals; however, those induced by EPEC are larger than the ones induced by the *esp*F mutant and this correlates with cellular ZO‐1 redistribution from the TJs; in the case of cells treated with the *espF* mutant, the pedestals are formed on the intercellular junctions. Since the pedestals are highly dynamic structure that allows the “surfing” of the bacteria along the cell (Shaner, Sanger, & Sanger, 2005), our data strongly suggest that TJ disassembly and the TJ proteins’ availability along the cytoplasm could be allowing their easy recruitment and thereby the pedestal movement along the cells, which could be relevant for infection spreading. The lack of TJ disassembly could avoid the movement of the pedestals along the cells, since, when EspF is lacking, these pedestals are formed on the intracellular junctions and are seen arrested there. Thus, we are still working for trying to demonstrate that EspF‐induced TJ disassembly and ZO‐1–afadin‐induced pedestal maturation (as shown here) could be required for pedestal movement. Interestingly, even though EspF is not required for pedestal formation (McNamara et al., 2001), ZO‐1 appears to be required for pedestal formation. The silencing of ZO‐1 results in a decrease in pedestal number, and pedestals are formed where residual ZO‐1 must be, particularly at sites of intercellular junctions. Moreover, these pedestals are smaller than those formed in ZO‐1‐enriched zones such as the cell–cell contacts or by ZO‐1 disassembly, either by EspF from EPEC or by using the calcium switch assay. It is noteworthy that ZO‐1 distribution is clearly homogenous along the pedestal structure when both EPEC infection and low‐calcium condition are applicated to epithelial cells as compared with cells infected with the *espF* mutant. These data suggest that EspF could be supporting a better distribution of ZO‐1 inside the pedestal. In fact, ZO‐1, as a protein connecting TJ membrane proteins to the actin cytoskeleton, exists in either stretched or folded conformations, ruled by actomyosin‐dependent force, resulting in changes in the localization, stability, and downstream signaling of its interactors (Spadaro et al., 2017). Additionally, it has been shown that ZO‐1 is incorporated within EPEC‐induced F‐actin bundles through its C‐terminal proline‐rich region (Hanajima‐Ozawa et al., 2007).

The eukaryotic linear motifs of EspF (proline‐rich sequences and the class III PDZ domain binding motifs) could be playing a role by interacting with actin binding proteins or scaffolding factors that recruit signaling molecules to cell junctions. Interestingly, EspF from EPEC 2348/69 (20.9 kDa) harbors three proline‐rich motifs and five class III PDZ domain binding motifs, whereas EHEC O157:H7 EspF (26.2 kDa) harbors the same motifs exactly in the same positions, but it is additionally extended by an proline‐rich motif and two PDZ domain binding motifs (Peralta‐Ramirez et al., 2008). However, it is well known that EPEC forms highest pedestals than EHEC (Shaner, Sanger, & Sanger, 2005). Thus, EspF motifs could be interfering with ZO‐1 and afadin, since ZO‐1 contains one SH3 and three PDZ domains and afadin contains three proline‐rich domains and one PDZ domain (Ooshio et al., 2010). Unlike EPEC, EHEC also expresses EspFU, which harbors five almost identical 47‐residue repeats (R47) consisting of 21% proline, including 22 putative SH3‐domain binding (PxxP) motifs. Thus, the C‐termini of EspF and EspFU are quite divergent; however, their similarity is because both are rich in proline residues (Campellone et al., 2004). EspFU is 25% identical to EspF and much of the homology between them extends over the first 60–70 residues (40% identical), but it is in the N‐terminus, which promotes type III translocation (Campellone et al., 2004). Unlike EspF, in the EspU R47_5_, the tandem PxxP motif is essential for the ability of EHEC to localize EspFU beneath bound bacteria and trigger the formation of an actin pedestal (Aitio et al., 2010). Thus, these proteins show different functions.

The relevant role of ZO‐1 for pedestal structure maturation led us to hypothesize that tight junctional proteins could be forming a TJ‐like complex between the eukaryotic membrane and the bacterial membranes. This hypothesis implies the participation of other proteins from the TJ such as transmembrane proteins, since ZO‐1 and ZO‐2 are cytoplasmic TJ proteins (Gumbiner, Lowenkopf, & Apatira, 1991). These latter proteins anchor actin filaments to membrane proteins through their C‐terminal regions, and the N‐terminal half of ZO‐1 binds to the TJ membrane proteins such as claudins and JAM (Ebnet, Schulz, Meyer Zu Brickwedde, Pendl, & Vestweber, 2000; Itoh et al., 1999). In order to explore the role of these tight junctional proteins, we used L cell cultures. These cells lack claudin (Furuse, Sasaki, Fujimoto, & Tsukita, 1998), occludin (Saitou et al., 1997), ZO‐2 and ZO‐3 (Itoh et al., 1999), and JAM‐B and JAM‐C (Morris et al., 2006), but express ZO‐1 (Itoh et al., 1993) and JAM‐A (Morris et al., 2006). Our data clearly show that EPEC is able to form pedestals in L cells similar to those formed in epithelial cells. Furthermore, pedestals formed in the cell–cell contacts were larger than those already formed along the cells; in L cells, these cell–cell contacts were enriched in ZO‐1. It has been reported that these nascent cell–cell contacts are primordial junctions where normally JAM‐A, ZO‐1, and PAR3–PAR6–aPKC complex are recruited (Zihni et al., 2016). In fact, we found that in L cells at 40% of confluence, the pedestals were smaller by avoiding these ZO‐1‐enriched zones in the cell–cell contact. All these data indicate that ZO‐1, but no other main TJ protein, is required for pedestal formation and growth. Moreover, unlike the primordial junctions, our results showed that JAM‐A is not recruited into the pedestals.

Even though no other main TJ proteins appear to be participating in pedestal maturation during EPEC infection, we decided to explore for another protein partner associated with ZO‐1 and in the pedestal maturation. Interestingly, L cells, which lack most of the intercellular junctional proteins, were still able to initiate these primordial junctions at the cell–cell contacts by enriching ZO‐1 in these sites. Indeed, it has been reported that in cells lacking TJs such as fibroblasts and astrocytes, ZO‐1 is localized at cell–cell contact sites with cadherin (Howarth, Hughes, & Stevenson, 1992; Itoh et al., 1993). Furthermore, afadin (Mandai et al., 1997) is localized with ZO‐1 at cell–cell contact sites in these types of cells (Yamamoto et al., 1997). The same authors found that afadin is colocalized with ZO‐1 at TJs of intestinal epithelial cells, whereas Ooshio et al. (2010) found that the formation of TJs in MDCK cells involves the interaction of afadin with ZO‐1. Here, we found that afadin colocalized with ZO‐1 at the pedestal structure, detected when cells were fixed with PFA 1% and then methanol–acetone, as used by Ooshio et al. (2010), instead of the classical fixation protocol using PFA 4%. Furthermore, it has been shown that afadin and ZO‐1 interact through PR1‐2 region of afadin recognizing the SH3 domain of ZO‐1 before the formation of TJs, whereas during and after the formation of TJs, ZO‐1 dissociates from afadin and associates with JAM‐A (Ooshio et al., 2010). Interestingly and similarly, in an EspF‐dependent form, EPEC recruits sequentially ZO‐1 and afadin at the pedestal structures, where both proteins interact before the pedestal growth. During the pedestal growth, ZO‐1 is dissociated from afadin, whereas after the pedestal maturation, afadin is recruited at the tip and ZO‐1 in the stem of the pedestals. In the TJ formation, ZO‐1 and afadin are required for JAM recruitment at the nectin cell–cell contacts (Fukuhara et al., 2002), but in pedestal maturation, JAM was not recruited in the pedestals enriched in ZO‐1 and afadin. In this way, these two main proteins necessary for the formation of TJs are required for pedestal maturation. Moreover, both are sequestrated in the pedestal structures, which must have strong consequences on the paracellular pathway of epithelia.

In fact, ZO‐1 and afadin are relevant for both TJ formation and pedestal maturation and ZO‐1 could also be important for pedestal formation. In the case of the intercellular junction, in ZO‐1 knockdown cells, afadin is not recruited to the TJ but to the adherent junctions (Ooshio et al., 2010). We found that in ZO‐1 knockdown cells, afadin is not recruited to the pedestal structure, and interestingly, the number of pedestals strongly decreased, suggesting that ZO‐1 is also necessary for pedestal formation. These data also suggest that besides Tir, which is critical for F‐actin recruitment to the pedestal, this process also requires ZO‐1 and afadin that are actin binding proteins, which could work together for forming these intracellular columns composed of an actin‐rich core region. In fact, it has been recently reported that multiple ZO‐1‐mediated interactions contribute to the coordination of epithelial actomyosin function and the genesis of unified apical surfaces. U5 and GuK domains of ZO‐1 are necessary for proper apical surface assembly, including organization of microvilli and cortical F‐actin (Odenwald et al., 2018). On the other hand, in afadin knockdown cells, ZO‐1 is not recruited to the TJ and proteins of the adherent junction are not either (Ooshio et al., 2010). Recently, these data have been confirmed in afadin knockout cells and it was shown that F‐actin‐binding (FAB) domain of afadin is also required for the formation of TJs (Sakakibara et al., 2018). We found that afadin is critical for pedestal maturation, and without this protein, the pedestals are smaller, but unlike ZO‐1, the lack of afadin did not decrease the number of pedestals per cell. Interestingly, the lack of afadin reallocates pedestal formation allowing that most of the pedestals were dispersed along the cells instead of in the cell–cell contacts. This suggests an initial recruitment of afadin to the nectin cell–cell contacts, as in the TJ formation, and then, further afadin recruitment induced by ZO‐1 occurs for the pedestal maturation process. Indeed, during EPEC infection in polarized cells, such as MDCK and HT29 cells, afadin is disassembled before ZO‐1 from the intercellular junctions, but afadin is recruited after ZO‐1 at the pedestal structures.

In conclusion, we have shown that ZO‐1 and afadin are recruited to the pedestal and they have a transient interaction leading to pedestal maturation. ZO‐1‐enriched zones are required for pedestal growth and for afadin recruitment. ZO‐1 and afadin transiently interact inside the pedestal structures that end with their dissociation and recruitment of afadin in the tip of the pedestal and ZO‐1 in the stem of these structures and finally leading to pedestal maturation. We speculate that the strategic localization of these proteins supports the coordination of epithelial actomyosin function that allows to maintain the intracellular columns composed of an actin‐rich core region, the pedestal structure. Additionally, this pedestal maturation could be also important for the dynamics of the pedestal movement along and between epithelial cells.

## 4 MATERIALS AND METHODS

### 4.1 Bacterial strains

The EPEC O157:H6 strain E2348/69 (Levine et al., 1978), EPECΔ*espF*, or EPECΔ*espF‐pespF* (in this work) was routinely grown in Luria–Bertani broth at 37°C overnight. Overnight cultures were activated for 2 hr in DMEM (Dulbecco's modified Eagle's medium) without antibiotics and serum at 37°C, as previously described (Rosenshine et al., 1996). When necessary, the medium was supplemented with kanamycin (50 µg/ml) and/or ampicillin (100 µg/ml).

### 4.2 *espF* deletion in EPEC E2348/69

To generate an *espF* deletion mutant of EPEC strain E2348/69, *espF* gene was replaced by a gene encoding kanamycin resistance by using the λ red recombinase system (Datsenko & Wanner, 2000). The kanamycin resistance gene was amplified from pKD4 by PCR with primers Fwr‐*espF*‐E23P (5′‐GAT ATA TAT GAG AGT TAG CCA AGA TTA GAT ATA AAG AGG CAT AAA TTT GTG TAG GCT GGA GCT GCT T‐3′) and Rev‐*espF*‐E23P (5′‐TTG GAA AAC AAA TAA TCA ATA CCG ATT AAT CGT TTT AAA TTA GTT GGT TAC ATA TGA ATA TCC TCC TTA G‐3′). The PCR‐generated products carrying the kanamycin resistance gene flanked by homologous regions of the chromosomal *espF* gene were treated with *Dpn*I and introduced into EPEC E2348/69, which was previously transformed with pKD46 expressing the λ red recombinase. Selection of EPEC∆*espF*::*kam* colonies was carried out as previously described (Datsenko & Wanner, 2000). *espF* deletion and *kam* insertion were verified by PCR. The EspF knockout was verified by Western blot using mouse specific antibodies against EspF.

### 4.3 *espF* cloning and complementation

The *espF* gene was amplified from genomic DNA of the prototypical strains EPEC E2348/69 using the primers C‐Fwr‐*espF* (5′‐ATG CTT AAT GGA ATT AGT AAC GCT GCT‐3′) and C‐Rev‐*espF* (5′‐CTC GAG CCC TTT CTT CGA TTG CTC ATA GGC‐3′). The PCR product, *espF*_EPEC_ (621 bp), was purified with QIAquick Gel Extraction Kit (Qiagen, Inc.) and cloned into pGEM‐T Easy vector (Promega Corp.) according to the manufacturer's instructions to create pGEMT‐*espF*_EPEC_. Subsequently, pGEMT‐*espF*_EPEC_ was digested with *Nco*I/*Xho*I and subcloned into the IPTG inducible vector pTrcHis2B (Thermo Fisher Scientific) to obtain the polyhistidine‐tagged *espF* fusion construction, pTrc‐*espF*_EPEC_His. For *espF* complementation, EPECΔ*espF* bacteria were transformed with pTrc‐*espF*_EPEC_His construction for subsequent infection assays.

### 4.4 Cell lines

MDCK II (Madin‐Darby canine kidney) and L cell fibroblasts (mouse subcutaneous connective tissue), generously provided by Dr. Lorenza González‐Mariscal Muriel (Department of Physiology, Biophysics and Neuroscience, Center for Research and Advanced Studies [CINVESTAV], Mexico City, Mexico), and HT‐29 human colorectal adenocarcinoma cell line (ATCC HTB‐38) were maintained in Dulbecco's modified Eagle's medium (DMEM) supplemented with 10% fetal bovine serum (Biowest, Nuaillé, France), 1% nonessential amino acids, 5 mM l‐glutamine, penicillin (100 U/ml), and streptomycin (100 µg/ml). All cell lines were incubated at 37°C in a humidified atmosphere at 5% CO_2_.

### 4.5 Infection assay

Overnight bacterial cultures grown in LB were diluted (1:20) and activated in DMEM without antibiotics or serum, and then incubated at 37°C until the mid‐log phase of growth was achieved. MDCK II, L, and HT‐29 cell lines were seeded on eight‐well Lab‐Tek chamber slides (Nalgene Nunc International, USA) or in 60‐mm Petri dishes. When cells reached a confluence of 95%, the monolayers were washed with antibiotic‐ and serum‐free DMEM and then infected with bacteria in DMEM to a multiplicity of infection (MOI) of 0.5 or 5 and maintained for the indicated times in a humidified incubator at 37°C and an atmosphere at 5% CO_2_.

### 4.6 Calcium switch assay

For Ca^2+^ switch experiments, MDCK and HT29 cells were seeded on eight‐well Lab‐Tek chamber slides and maintained in DMEM supplemented with 10% fetal bovine serum, 1% nonessential amino acids, 5 mM l‐glutamine, penicillin (100 U/ml), and streptomycin (100 µg/ml). After 48 hr of culture, cells at 95% of confluence were used for calcium switch assay as follows: For normal‐calcium condition, cells were washed with antibiotic‐ and serum‐free DMEM containing a normal calcium concentration (1.8 mM Ca^2+^) and starved in the same culture medium for 2 hr. For low‐calcium condition, cells from normal‐calcium condition (after 2 hr) were washed with antibiotic‐ and serum‐free DMEM without calcium (DMEM; Gibco 21068–028), which was measured with a Ca^2+^‐sensitive electrode to contain 1–5 µM Ca^2+^ (Gonzalez‐Mariscal et al., 1990), and starved in the same culture medium for 2 hr. For recovery condition, cells from low‐calcium condition (after 2 hr) were washed with antibiotic‐ and serum‐free DMEM containing normal‐calcium condition and maintained in the same culture medium for 2 hr. For each condition, cells were infected with EPEC or EPEC∆*espF* for the indicated times.

### 4.7 Confocal microscopy

Eight‐well Lab‐Tek chamber slides (Nalgene Nunc International) were seeded with MDCK II, L, or HT‐29 cell lines. For immunofluorescence assay, MDCK II (95% of confluence), L (80 or 40% of confluence), and HT‐29 (95% of confluence) cells were seed at the indicated confluences. Cells were washed with antibiotic‐ and serum‐free DMEM and starved in the same culture medium for 2 hr. After that, cells were infected with EPEC, EPEC∆*espF*, or EPEC∆*espF‐pespF* for the indicated times. Infected cells were gently fixed in two steps: with 1% PFA for 15 min at room temperature (to fix pedestal structures), washed seven times with cold PBS, and then, cells were fixed and permeabilized with a mixture of 50% acetone and 50% methanol at −20°C for 7 min, and subsequently, cells were washed seven times with cold PBS. Fixed cells were blocked with 1% BSA in PBS for 1 hr. Slides were incubated overnight at 4°C with the different primary antibodies used: rabbit anti‐ZO‐1 polyclonal antibody (Thermo Fisher Scientific), mouse anti‐ZO‐1 monoclonal antibody (Thermo Fisher Scientific), or rabbit anti‐afadin polyclonal antibody (Sigma‐Aldrich; Merck KGaA), or rabbit anti‐JAM‐A polyclonal antibody (Thermo Fisher Scientific). After incubation with the primary antibodies, slides were incubated for 1 hr at room temperature with the following secondary antibodies: FITC‐goat anti‐rabbit (Thermo Fisher Scientific), biotin‐SP‐conjugated AffiniPure goat anti‐rabbit IgG (JIR) and then dichlorotriazinylamino fluorescein (DTAF)‐conjugated streptavidin (JIR), or CY5‐donkey anti‐mouse IgG (JIR). Slides were also alternatively stained for actin stress fibers and actin pedestals by using tetramethyl rhodamine isothiocyanate–phalloidin (Molecular Probes‐Invitrogen), and cell and bacterial DNA were stained using DAPI (2‐(4‐amidinophenyl)‐6‐indolecarbamidine dihydrochloride, 4′,6‐diamidino‐2‐phenylindole dihydrochloride) (Sigma‐Aldrich; Merck KGaA). The samples were mounted with VECTASHIELD (Vector Laboratories) and analyzed using a Leica TCS SP8 confocal microscope (Leica Microsystems).

### 4.8 Quantimetric analyses of confocal microscopy images

Pedestal size was measured by detecting the F‐actin signal of these structures and using 150 pedestal structures from 5 field images for each experiment (*n* = 3) using LAS AF Lite (Leica Application Suite Advanced Fluorescence) software. Each pedestal was measured using 0.5‐μm optical slices to allow individual pedestal measurement, and they were measured from the base of the pedestal (start of the stem) to the top of the pedestal (end of the tip), where the bacteria were adhered. Pedestal data were plotted as the mean size of each treatment and their standard error to the mean (means ± *SEM*). Comparisons between groups were made using one‐way analysis of variance (ANOVA) with Tukey's multiple comparison.

Protein recruitment into the pedestal structures was measured by detecting the fluorescence signal (ZO‐1 in red and afadin in green), which was delimited as the region of interest (ROI) in 150 pedestals as mentioned above (*n* = 3 independent experiments), using the Fiji 2.1.0 (ImageJ) software.

Colocalization between ZO‐1 and afadin in the pedestals was measured by delimiting the region of interest of pedestal structures in 150 pedestals, using the Fiji 2.1.0 (ImageJ) software. The Pearson correlation coefficients for ZO‐1 and afadin colocalization in these structures were plotted as the means of these coefficients and their standard error to the mean (*n* = 3 independent experiments).

For pedestal quantification, the number of pedestals in the three maturation phases was quantified in at least 5 optical fields (63× zoom) for each group, using the LAS AF Lite software. The means ± *SE* of the pedestal number by optical field were plotted and compared as mentioned above (*n* = 3 independent experiments).

### 4.9 Co‐immunoprecipitation assays

L cells were seeded on 60‐mm Petri dishes and incubated at 37°C in a humidified atmosphere at 5% CO_2_ until 95% of confluence was reached. Then, cells were infected as described above with EPEC or EPEC∆*espF* at a MOI of 5 for the indicated times. Proteins were co‐immunoprecipitated using protein A‐agarose according to the manufacturer's instructions (Roche, Mannheim, Germany). Briefly, uninfected (mock) and infected cells were washed three times with cold PBS and scraped into 500 µl of RIPA lysis buffer (50 mM Tris‐HCl [pH 7.5], 150 mM NaCl, 1% IGEPAL, 0.5% sodium deoxycholate), supplemented with cOmplete^™^ protease inhibitor cocktail (Roche), and sonicated in cold for 10 s at 30% amplitude. Samples were kept in lysis buffer in rotary agitation at 4°C for 20 min. A fraction was used for protein quantification. Then, samples containing 750 μg proteins were incubated with 1 µg of rabbit anti‐ZO‐1 polyclonal antibody (Thermo Fisher Scientific, Waltham, MA, USA) in rotary agitation at 4°C overnight. Immunocomplexes were captured using 25 µl of protein A‐agarose beads for 3 hr at 4°C in rotary agitation. Agarose–antibody–antigen complexes were centrifuged for 1 min at 1,000 rpm. Supernatants were discarded and pellets were washed with 1 ml of RIPA lysis buffer and centrifuged for 1 min at 1,000 rpm. Pellets were washed twice with 1 ml of high‐salt buffer (50 mM Tris‐HCl [pH 7.5], 500 mM NaCl, 0.1% IGEPAL, 0.05% sodium deoxycholate) and centrifuged for 1 min at 1,000 rpm. Then, pellets were washed with 1 ml of low salt buffer (50 mM Tris‐HCl [pH 7.5], 0.1% IGEPAL, and 0.05% sodium deoxycholate) and centrifuged for 1 min at 1,000 rpm. Agarose pellets were suspended in 30 µl of loading buffer and boiled for 10 min. Proteins were separated by SDS‐PAGE and transferred to PVDF membranes and analyzed by Western blot using antibodies rabbit anti‐ZO‐1 polyclonal antibody, rabbit anti‐afadin polyclonal antibody, and mouse anti‐EspF polyclonal antibody. Protein bands were analyzed by densitometry. Densitometric analyses were performed using the Fiji 2.1.0 software. All data were analyzed and plotted using the GraphPad Prism 6.0 software. Data are expressed as means ± *SE* of three independent experiments, and comparisons between groups were made using one‐way analysis of variance (ANOVA) with Tukey's multiple comparison (*p* ≤ .05).

### 4.10 RNA interference

L cells were seeded on 35‐mm Petri dishes at 50% of confluence in DMEM supplemented with 10% fetal bovine serum, 1% nonessential amino acids, 5 mM l‐glutamine, penicillin (100 U/ml), and streptomycin (100 µg/ml) and incubated at 37°C in a humidified atmosphere at 5% CO_2_. After 18 hr, cells were washed two times with antibiotic‐ and serum‐free advanced RPMI 1640 medium (Thermo Fisher Scientific) and incubated in the same medium for 1 hr. ZO‐1 knockdown was performed by transiently transfected cells with a mixture containing 5 µg of mZO1‐1 shRNA, a gift from Alan Fanning (Addgene plasmid # 37215), plus 5 µl of Lipofectamine 2000 (Thermo Fisher Scientific) or the empty vector pLL5.0 (kindly provided by Christina Van Itallie and James Anderson from The Laboratory of Tight Junction Structure and Function, National Heart, Lung, and Blood Institute, National Institutes of Health), plus 5 µl of Lipofectamine 2000. When the ZO‐1 knockdown was performed in L cells grown on eight‐well Lab‐Tek chamber slides, 1 µg of mZO1‐1 shRNA or pLL5.0 plus 1 µl of Lipofectamine 2000 was used.

Afadin knockdown was performed by transfecting L cells with a mixture containing 70 pmol of Silencer Select predesigned siRNA (afadin siRNA) for murine afadin (Silencer; Thermo Fisher Scientific, siRNA ID 501170) plus 5 µl of Lipofectamine 2000, or the control scramble siRNA‐A (Santa Cruz Biotechnology) plus 5 µl of Lipofectamine 2000. When the afadin knockdown was performed in L cells grown on eight‐well Lab‐Tek chamber slides, 12 pmol of afadin siRNA or scramble siRNA plus 1 µl of Lipofectamine 2000 was used. In order to select positively transfected cells, a plasmid encoding the green fluorescent protein (GFP; pGreen Lantern‐1; Gibco BRL) was cotransfected with both synthetic siRNAs. After 72 hr, ZO‐1 or afadin knocked down or control transfected cells were harvested in lysis buffer, and relative ZO‐1, afadin, and actin levels were analyzed by Western blot. Alternatively, knockdown cells were infected with EPEC for 4 hr and then analyzed by confocal microscopy. The number of pedestals was determined using LAS AF Lite software by analyzing at least 30 transfected cells in each experiment. Means ± *SE* were plotted, and comparisons between groups were made using one‐way analysis of variance (ANOVA) with Tukey's multiple comparison.

## CONFLICT OF INTERESTS

The authors confirm that this article content has no conflict of interest.

## AUTHOR CONTRIBUTION

FNG and PUS conceived and designed the study; performed analysis and interpretation of the data, and wrote the manuscript; and PUS performed the experiments.

## ETHICS STATEMENT

None required.

## Data Availability

All data are provided in full in the Results section of this paper.
